# Decoupling Host Preference and Performance in *Callosobruchus maculatus* (Fabricius, 1775): Roles of Seed Biochemistry and Botanical Insecticides in Stored Legumes

**DOI:** 10.3390/insects17070671

**Published:** 2026-06-26

**Authors:** Rasheed Akbar, Gul Makai, Rehan Kausar, Ambreen Ijaz, Brekhna Faheem, Naseem Rafiq, Shehreyar Javed, Imtiaz Ali Khan, Jibiao Fan, Jianfan Sun

**Affiliations:** 1 College of Animal Science and Technology, Yangzhou University, Yangzhou 225009, China; rasheed.akbar@uoh.edu.pk; 2 Department of Entomology, Faculty of Physical and Applied Sciences, The University of Haripur, Haripur 22062, Khyber Pakhtunkhwa, Pakistan; 3 Department of Zoology, Sardar Bahadar Khan Women University, Quetta 86400, Balochistan, Pakistan; 4 Department of Statistics, Sardar Bahadar Khan Women University, Quetta 86400, Balochistan, Pakistan; 5 Department of Zoology, Abdul Wali Khan University Mardan, Mardan 23200, Khyber Pakhtunkhwa, Pakistan; 6 Department of Entomology, The University of Agriculture Peshawar, Peshawar 22062, Khyber Pakhtunkhwa, Pakistan; 7 Institute of Environment and Ecology, School of Environment and Safety Engineering, Jiangsu University, Zhenjiang 212013, China; 8 Jiangsu Collaborative Innovation Center of Technology and Material of Water Treatment, Suzhou University of Science and Technology, Suzhou 215009, China

**Keywords:** *C. maculatus*, host preference, seed biochemistry, botanical insecticides, *A. indica*, stored product protection

## Abstract

*Callosobruchus maculatus* (Fabricius, 1775) is a major postharvest pest of stored legumes, causing significant quantitative and qualitative losses. This study evaluated host preference, biochemical determinants of susceptibility, and the insecticidal efficacy of plant powders and aqueous and ethanolic extracts against *C. maculatus* among selected pulse species. In free-choice assays, oviposition preference did not correspond with developmental success, as *Phaseolus vulgaris* Linnaeus, 1753, received the highest number of eggs but supported minimal adult emergence, whereas *Vigna radiata* (Linnaeus) A. Jussieu, 1954, exhibited the highest level of infestation and seed damage. Biochemical analysis revealed correlational patterns: higher carbohydrate and lipid contents were positively associated with infestation and seed weight loss, whereas fiber content showed a negative association with pest performance. Protein content was correlated with oviposition, while moisture and fiber were associated with reduced developmental success. Plant-derived treatments significantly affected all biological parameters of *C. maculatus* in a concentration- and time-dependent manner, with the exception of sex ratio. Among the tested botanicals, *Azadirachta indica* A. Jussieu (1830) consistently showed the highest toxicity, with the lowest LC_50_ values across exposure periods. Ethanolic extracts showed greater insecticidal activity than aqueous extracts, indicating solvent-dependent extraction of bioactive compounds. Mortality increased progressively with exposure duration, accompanied by decreasing LC_50_ values, suggesting cumulative toxic and developmental effects. Overall, the results demonstrate that host suitability is governed by the interaction between nutritional composition and structural seed traits, while botanical extracts, particularly those from *A. indica*, offer effective, eco-friendly alternatives for managing *C. maculatus* in stored legumes.

## Simple Summary

This study evaluated host preference, biochemical determinants, and botanical insecticidal efficacy against the major postharvest pest *Callosobruchus maculatus* (F.) in stored legumes. Oviposition preference did not match developmental success: *Phaseolus vulgaris* received the most eggs but exhibited low adult emergence, while *Vigna unguiculata* exhibited the highest level of infestation. Biochemical analysis revealed that higher carbohydrate and lipid contents were positively associated with infestation and seed weight loss, whereas fiber content negatively affected pest performance. Protein content was linked to oviposition, while moisture and fiber reduced developmental success. Among plant-derived treatments, *Azadirachta indica* exhibited the highest toxicity (lowest LC_50_), with ethanolic extracts more effective than aqueous ones. Mortality increased with exposure time, indicating cumulative toxic effects. Host suitability is governed by nutritional composition and seed traits, and botanical extracts, especially from *A. indica*, offer effective, eco-friendly management options.

## 1. Introduction

Postharvest losses caused by stored-product insect pests remain a persistent threat to global food security, especially in legumes, which constitute a main source of dietary protein in many developing regions [1]. Among these pests, bruchid beetle, *Callosobruchus maculatus* (F.) (Coleoptera: Chrysomelidae: Bruchinae), is a highly destructive species that causes severe damage to stored pulses [2]. Typically, infestation begins in the field and continues during storage, during which rapid population growth, higher reproductive capacity, and concealed larva development within seeds makes control difficult and economically costly [3]. Under traditional storage conditions, losses in susceptible legumes can exceed 30–60%, depending on host suitability and storage conditions [4,5]. The pests can produce several generations during storage within a relatively short time, further exacerbating postharvest losses and reducing seed viability, market value, and nutritional quality [6].

The biology of *C. maculatus* is strongly affected by host seed characteristics, especially those associated with nutritional composition and physical structure [7]. Females oviposit on the surface of the host seed, and, after hatching, the larvae penetrate the seed coat and complete their development inside the seed, where they are concealed from external control measures [8]. Host suitability is determined by different factors such as oviposition preference, larval survival, developmental rate, and successful emergence of adult beetles [9]. Earlier research has shown that oviposition choice is not always corroborated with ideal developmental conditions, giving rise to a well-documented preference–performance mismatch in bruchid systems [10,11]. This mismatch revealed that female beetles may rely on unreliable or incomplete cues when selecting oviposition sites, leading to maladaptive reproductive decisions under varying host availability conditions.

In stored pulses and legumes, host plant resistance has been attributed to both physical and biochemical seed traits. Carbohydrates, proteins, and lipids are the nutritional components that may encourage larval growth by providing free energy and metabolic substrates [9]. On the other hand, larval penetration and developmental success are inhibited by structural components including fiber content, seed coat thickness, and seed hardness [12]. Also, different secondary metabolites such as phenolics, tannins, and protease inhibitors have been associated with antibiosis-based resistance mechanisms that negatively affect insect digestion, growth, and survival [13,14]. However, among different legumes species, the relative contributions of these traits differ, and their interactive effects on *C. maculatus* performance remain insufficiently understood [7]. Notably, associations between insect performance and seed composition do not necessarily imply direct causation, as several biochemical characteristics may be confounded with structural and genetic factors that influence seed development.

Among the economically significant crops, leguminous hosts seeds such as *Vigna unguiculata* (L) Walpers, 1842, *Vigna radiata* (L.), *Pisum sativum* Linnaeus, 1753, *Phaseolus vulgaris* L., and *Cicer arietinum* Linnaeus, 1753, have shown varying degrees of susceptibility to bruchid beetle infestation [9,15]. As a recognized susceptible host to *C. maculatus*, cowpea is a widely preferred laboratory host because it supports quick insect development [16]. On the other hand, common beans and other legumes may show partial resistance, despite sometimes receiving maximum oviposition [17]. The complexity of host–pest interactions highlights that resistance may be expressed at post-ovipositional stages rather than by oviposition deterrence [18]. Identifying the host traits that confer resistance and understanding the biochemical basis of differences in these traits are essential for developing resilient pest management strategies.

The increasing limitations of synthetic insecticides in stored-product protection, including resistance development, environmental contamination, and concerns regarding consumer health, have encouraged interest in plant-derived alternatives [19]. Owing to their complex modes of action, biodegradability, and reduced risk of resistance development, plant-based biocides represent a promising category of biorational control agents [20]. Crude plant extracts and plant powders have been extensively used against storage pests, with varying degrees of success depending on the plant species, extraction method, and target insect pest [14]. The modes of action of these plant products include feeding inhibition, oviposition deterrence, growth disruption, and direct toxicity, often mediated by secondary metabolites such as alkaloids, terpenoids, flavonoids, and limonoids [21].

*Azadirachta indica* (neem) A. Jussieu (1830) plant extract is a plant-derived insecticide that has been thoroughly studied because of its broad-spectrum activity and multiple modes of action [22]. Azadirachtin and related limonoid compounds present in *A. indica* are known to disrupt insect endocrine regulation, especially ecdysteroid-mediated molting processes, resulting in abnormal development and mortality [23]. Similarly, nicotine, which is present in tobacco plants, acts on nicotinic acetylcholine receptors and causes neurotoxic effects in insect pests [24]. Several other aromatic and medicinal plant species contain different secondary metabolites that have antifeedant and repellent effects [25]. The efficacy of these plant-based materials varies significantly depending on the extraction method, type of solvent, polarity, exposure time, and concentration, showing that comparative evaluations must be performed under standardized conditions [26].

Despite extensive research on plant-based insecticides and *C. maculatus*–host interactions, a knowledge gap remains regarding the integrated relationship among insect activity, host seed composition, and plant-based biocide control efficacy. First, most research has focused either on host preference or on toxicity assays, with limited integration of biochemical host profiling and insect biological responses. Second, the relative importance of structural seed traits versus nutritional composition in determining susceptibility remains unknown. Third, under free-choice infestation conditions, comparative assessments of plant powders and extracts of plant species across several exposure durations and biological endpoints are still limited. These gaps limit the development of predictive frameworks for host susceptibility and botanical control effectiveness.

Therefore, this study was designed to examine the interaction between *C. maculatus* performance and biochemical composition across different legume species and to investigate the effects of selected plant species in powder and extract form against various parameters of *C. maculatus*. Specifically, this study aimed to (i) examine oviposition preference and developmental success across selected pulse hosts, (ii) determine the relationship between pest infestation parameters and seed biochemical composition, and (iii) evaluate the concentration- and time-dependent effects of plant powders and aqueous/ethanolic extracts on mortality, reproduction, and seed damage. By integrating host suitability analysis with botanical insecticide evaluation, this research presents a comprehensive framework for understanding both ecological interactions and applied pest management potential in stored legume systems.

## 2. Materials and Methods

### 2.1. Insect Rearing Conditions

The population of *C. maculatus* (F.) was established in the entomological laboratory at the University of Haripur Pakistan. Adult beetles were collected from infested cowpea (*Vigna unguiculata*) seeds obtained from a local market in Haripur in 2025. The colony was maintained on certified insecticide-free cowpea seeds in 2 L glass jars covered with fine mesh cloth for ventilation. Rearing conditions were set at 28 ± 2 °C, relative humidity of 65 ± 5%, and a photoperiod of 12:12 h (light:dark). Fresh *C. maculatus* adults (24 h old) were used for all experiments [27]. To ensure genetic homogeneity, the colony was refreshed every three generations with field-collected individuals.

### 2.2. Host Legume Species

Five legume species were obtained from the same local market and stored at 4 °C until use. The seeds were surface-sterilized by brief immersion in 1% sodium hypochlorite solution for 2 min, followed by three rinses with distilled water and air drying. The species were *P. sativum* L.; *C. arietinum* L.; *P. sativum* L.; *V. radiata* L. R. Wilczek; and *V. unguiculata* L. Walp. Only undamaged, uniformly sized seeds were used. Before the experiments, seed moisture content was equilibrated to 12 ± 1% by storing at 25 °C for 1 week.

### 2.3. Host Preference Bioassay (Free-Choice Test)

The free-choice oviposition and development test followed the methods of Raina [28]. Fifty grams of each legume species were placed in separate, labelled plastic containers (15 cm diameter × 10 cm height) arranged randomly inside a larger experimental arena (60 cm × 60 cm × 40 cm) with a nylon mesh top. Ten pairs (10♂:10♀) of newly emerged beetles of the same age and size were released into the test arena and allowed to lay eggs for 72 h. Then, the released adults were removed, and four replications were allowed to occur. The parameters recorded after oviposition are described in [22]. It is important to note that equalizing seed mass (50 g per legume species) does not control for inter-species differences in seed size, shape, surface area, or number per gram. Therefore, the number of eggs recorded per host mass reflects a combination of female behavioral choice and the morphological characteristics of the seeds. The term “oviposition preference” is used here operationally to denote egg distribution under free-choice conditions, not as a pure measure of behavioral preference independent of seed morphology.

**Number of eggs**: We randomly selected 50 seeds of each host species and manually counted them using a stereomicroscope (10×).
**Adult emergence (%)**: After oviposition had occurred and 28 days had elapsed (sufficient time for complete development), emerged adults were counted on a daily basis for 10 consecutive days [29].
  $$Percentage\;emergence=\frac{Number\;of\;emerged\;adults}{Number\;of\;eggs}\times 100$$
**Infestation rate (%)**: The seeds which showed emergence holes were defined as infested seeds, and the following formula was used to calculate the percent infestation rate [30].
  $$Infestation\;rate(\%)=\frac{Infested\;seeds}{Total\;seeds}\times 100$$
**Seed weight loss (%)**: The weight of host seeds before and after the experiment (after removing adults and frass) was measured [22].
  $$Host\;Seed\;Weight\;loss(\%)=\frac{\left(Initial\;weight-Final\;weight\right)}{Initial\;weight}\times 100$$
**Sex ratio**: Male and female adult beetles were separated based on physical appearance, i.e., female beetles are large and have a plate at the end of the abdomen, while male beetles are small, and the plate at the end of the abdomen is absent [31].

### 2.4. Biochemical Analysis of Legume Seeds

For biochemical analysis of each legume species, 100 g of clean, undamaged host seed were ground into a fine powder using a laboratory mill (Cyclotec 1093, Foss, Höganäs Sweden). An air-tight glass vial was used to store the powder at room temperature until analysis, and all analyses were conducted three times [32].

The proximate composition of the samples was assessed using standard AOAC methods [33]. To determine the moisture content, the host seed samples were dried in an oven at 105 °C for 24 h (AOAC Method 925.10). To measure the ash content, the host seed samples were burned in a muffle furnace at 550 °C for 6 h (AOAC Method 923.03). The Kjeldahl method with a conversion factor of 6.25 was used to determine the crude protein content (AOAC Method 984.13). Crude fat was measured by Soxhlet extraction using petroleum ether (40–60 °C) for 8 h (AOAC Method 920.39). Crude fiber was determined by acid–alkali digestion using a Fibertec system (Foss, Hillerød, Denmark) according to AOAC Method 962.09. The carbohydrate content (100 − (moisture + ash + protein + fat + fiber) was determined on a dry weight basis. For measurement of pH, 5 g of host seed powder was mixed with 25 mL of distilled water and stirred for 30 min, and the pH was measured using a calibrated pH meter (Mettler-Toledo, Columbus, OH, USA).

### 2.5. Plant Materials for Botanical Treatments

Five plant species were selected based on their common use. These were *A. indica* A. Juss. leaves, *Nicotiana tabacum* (L.) dried leaves, *Nicotiana rustica* (L.) dried leaves, *Melia azedarach* (L.) leaves, and *Thuja orientalis* (L.). Plant materials were shade-dried at 25–30 °C for 14 days, then ground to a fine powder (passed through a 0.5 mm mesh size sieve) using an electric grinder. Powders were stored in sealed amber glass bottles at 4 °C until use [34]. *Nicotiana* was included specifically for comparative phytochemical screening and is not proposed as a practical control agent.

### 2.6. Preparation of Plant Extracts

#### 2.6.1. Aqueous Extract

Fifty grams of each plant powder were mixed with 500 mL of distilled water (1:10 *w*/*v*) in a conical flask and shaken on an orbital shaker (150 rpm) for 24 h at room temperature. The mixture was filtered through double-layered muslin cloth, followed by filtration through Whatman No. 1 filter paper. The filtrate was concentrated under reduced pressure at 40 °C using a rotary evaporator (Meierseggstrasse 40, Postfach 9230, Flawil, Switzerland). The resulting extract was stored at 4 °C [14]. For bioassays, stock solutions were freshly prepared in distilled water and diluted to the required concentrations (0.5%, 1.0%, 1.5%, 2.0%, 2.5%, 3.0% *w*/*v*).

#### 2.6.2. Ethanolic Extract

Fifty grams of plant powder were extracted with 500 mL of 95% ethanol (1:10 *w*/*v*) in a Soxhlet apparatus for 8 h. The extract was concentrated under reduced pressure at 40 °C to dryness and stored at 4 °C [35]. For residual toxicity assays, the extract was dissolved in ethanol and diluted to the same concentration range as above (0.5–3.0% *w*/*v*). The control contains only 95% ethanol (ethanolic), without plant material.

For aqueous extracts, the concentrated residue was weighed, and the extraction yield was calculated as (dry extract weight/initial plant powder weight) × 100%.

For ethanolic extracts, the dried extract was weighed, and the yield was recorded similarly.

#### 2.6.3. Powder Treatment

For this experiment, plant species powders were mixed with clean *V. unguiculata* seeds (susceptible standard) at rates of 0.5, 1.0, 1.5, 2.0, 2.5, and 3.0% (*w*/*w*). These concentrations were used directly. Treated seeds were tumbled in sealed plastic bags for 10 min to ensure uniform mixing [36].

### 2.7. Biological Response Bioassays (Oviposition, Emergence, Infestation, Weight Loss, and Sex Ratio)

A series of concentration-response experiments for each plant species and each extract type (aqueous extract, ethanolic extract, and plant powder) was conducted using the susceptible host *V. unguiculata* (L.) Walp [36]. Twenty grams of seeds treated with extract solution and then air-dried were placed in glass jar 10 cm diameter. Ten newly emerged pairs (10♂:10♀) of beetles of the same age and size were released and allowed to oviposit for 72 h. The same parameters as in Section 2.3, number of eggs, % adult emergence, % infestation, % seed weight loss, and sex ratio, were recorded after adult removal. Each treatment (plant species × concentration) was replicated four times, along with a control. For aqueous and ethanolic extracts, distilled water and ethanol were used as solvent controls, respectively. These controls were also assayed and exhibited no significant difference from untreated controls. The data were pooled.

### 2.8. Toxicity Bioassays (Mortality)

Contact toxicity (for aqueous extracts) and residual toxicity (for ethanolic extracts and powders) were evaluated separately. In all cases, 20 unsexed, 0–24 h old adult weevils were placed in each container (10 cm diameter) containing 20 g of treated seeds. Mortality was recorded at 24, 48, 72, and 96 h after exposure. Weevils were considered dead if no movement was observed after gentle prodding. Each concentration was tested five times, and controls (untreated seeds) were included for each time point. Mortality in controls was always <5% and was corrected using the Schneider Orelli (1947) formula [37]:

$$Corrected\;mortality(\%)=\frac{\left(Treatment\;mortality-Control\;mortality\right)}{\left(100-Control\;mortality\right)}\times 100$$

The choice of toxicity assay differed by formulation type based on their physical properties. Aqueous extracts were tested using a contact toxicity method, because water-based solutions readily coat seed surfaces and come into direct contact with weevils upon introduction. In contrast, ethanolic extracts and plant powders were evaluated using a residual toxicity method, as ethanol evaporates quickly, leaving behind bioactive residues on seed surfaces, and powders persist as physical deposits. Consequently, the LC_50_ values of aqueous extracts (contact) and ethanolic extracts or powders (residual) are not directly comparable in absolute terms, because the routes and durations of exposure differ. Comparisons across formulation types should therefore focus on relative trends within each assay rather than absolute potency rankings.

### 2.9. Statistical Analysis

All data were tested for normality (Shapiro–Wilk test) and for homogeneity of variances (Levene’s test). Percentage data (adult emergence, infestation, weight loss, sex ratio, mortality) were arcsine square root-transformed before analysis, when necessary, but untransformed means are presented in the results. Two-way analysis of variance (ANOVA) was used to test the effects of plant species, concentration, and their interaction on each response variable [38]. Where significant interactions were detected, post hoc comparisons were performed using Tukey’s HSD test (α = 0.05). For host preference data (Section 3.1), one-way ANOVA followed by Tukey’s test was used to compare legume species [39,40]. Pearson’s correlation coefficients (r) were calculated, and significance was set at *p* < 0.05 [41,42,43]. All statistical analyses were performed using R (version 4.2.0). Figures (heatmaps, dendrograms, dose–response curves) were generated using the ggplot2 and heatmap packages in R.

## 3. Results

### 3.1. Host Preference of C. maculatus (F.) Among Different Pulses

In the free-choice test, all grain types were simultaneously exposed to infestation by *C. maculatus*, following the methods of Raina [28]. Significant differences were observed among host species in terms of oviposition preference, adult emergence, infestation rate, and seed weight loss (Table 1). Because equal seed mass does not control for differences in seed size, surface area, or number, the oviposition data reflect a combination of female behavioral responses and seed morphological traits. Oviposition preference differed significantly (F = 124, *p* < 0.0001), with the highest number of eggs laid on *P. vulgaris* L. (166.80 ± 1.34) and the lowest on *P. sativum* (88.30 ± 1.34). However, adult emergence showed an opposite trend (F = 232, *p* < 0.0001), with the highest emergence on *V. radiata* (L.) R. Wilczek (96.00 ± 0.46%) and the lowest on *P. sativum* L. (4.00 ± 0.33%), indicating that oviposition preference did not correspond to successful development. Infestation rate was also significantly affected (F = 115, *p* < 0.0001), being highest on *V. unguiculata* (L.) Walp. (1842) (69.00 ± 1.43%) and lowest on *P. sativum* (3.00 ± 0.59%). A similar pattern was observed for seed weight loss (F = 109, *p* < 0.0001), with the greatest loss in *V. unguiculata* (53.00 ± 0.94%) and the least in *P. sativum* (2.00 ± 0.31%). In contrast, the female-to-male sex ratio did not differ significantly among the five host species. Although a slightly higher proportion of females (50.40 ± 2.60%) than males (49.60 ± 2.60%) was recorded on *P. sativum*), males were marginally more abundant on the other hosts Tables S1–S6.

### 3.2. Biochemical Analysis

Hierarchical clustering of standardized compositional data (z-scores) revealed substantial inter-species variation across the seven evaluated nutritional parameters (carbohydrates, fats, pH, proteins, moisture, ash, and fiber). Among host seed species, heatmap values ranged from −1.52 to 1.66 standard deviations, showing prominent divergence. The dendrogram showed two primary clusters, with *P. sativum* grouping closely with *C. arietinum* L., while *P. sativum* L., *V. radiata*, and *V. unguiculata* formed a distinct cluster. The proximity of *P. sativum* and *C. arietinum* shows relatively similar compositional profiles, especially with respect to ash and fiber content, whereas the second cluster revealed greater internal variability.

**Figure 1:**
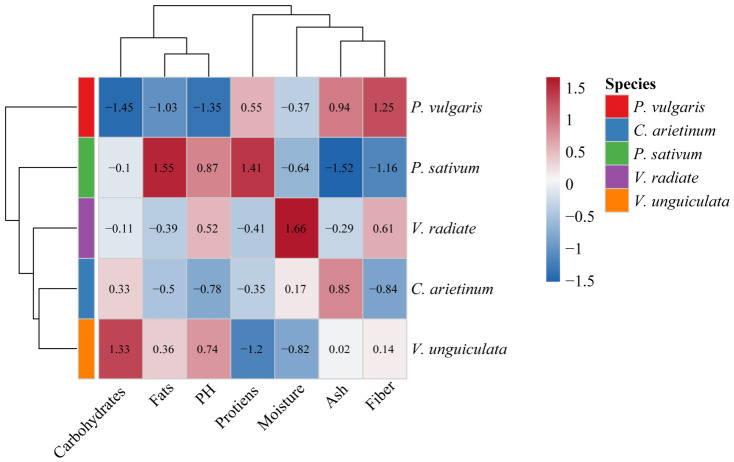
Clustered heatmap showing normalized nutritional composition (carbohydrates, fats, pH, proteins, moisture, ash, and fiber) across five legume species (*P. vulgaris*, *C. arietinum*, *P. sativum*, *V. radiata*, and *V. unguiculata*. Data represent means of four replicate biochemical analyses (*n* = 4 per species). Values were z-score-standardized by trait. Hierarchical clustering was performed using Euclidean distance and Ward’s linkage method in R (version 4.2.0; heatmap package). The dendrograms show similarity among species (left) and among nutritional traits (top). Color scale ranges from −1.52 (low) to 1.66 (high) standard deviations.

Comparison among the seed hosts’ trait-specific patterns further highlighted distinct compositional signatures. Carbohydrate content was maximum in *V. unguiculata* (z = 1.33) and lesser in *P. sativum* (z = −1.45), revealing clear variation in energy storage components. The fat (z = 1.55) and protein (1.41) content of *P. sativum* distinguished it from the other seed species (Figure 1). The moisture content was lower (z = −0.82) in *V. unguiculata* and higher in *V. radiata* (z = 1.66). In *P. vulgaris* (z = 0.94), the ash content was relatively enriched, followed by *C. arietinum* (z = 0.85), revealing differences in mineral-associated fractions. The fiber content was reduced in *P. sativum* (z = −1.16) and *C. arietinum* (z = −0.84) and highest in *P. sativum* (z = 1.25). Across the host seed species, differences in pH were comparatively moderate and did not strongly contribute to cluster separation. Overall, the clustering pattern shows that species differentiation is driven by the combined contribution of multiple compositional traits rather than any single variable, with clear evidence of multivariate divergence among the studied legumes.

### 3.3. Correlation Among Biochemical Contents and C. maculatus Oviposition, Adult Emergence, Host Infestation, and Host Seed Weight Loss

**Figure 2:**
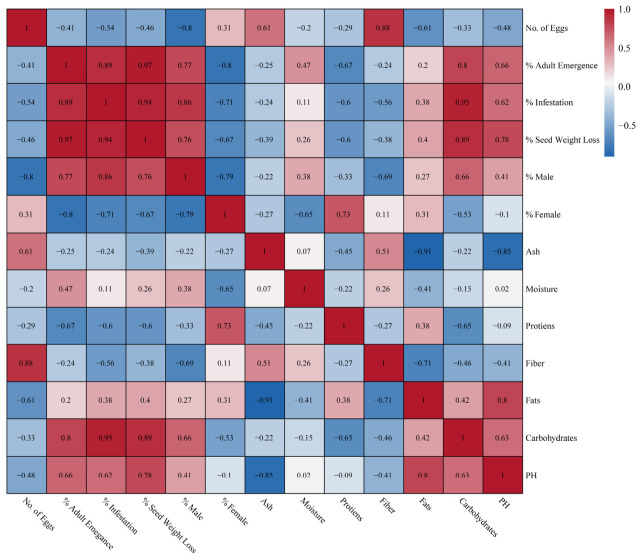
Pearson correlation matrix showing the relationships between pest damage parameters (number of eggs, % adult emergence, % infestation, % seed weight loss, % male, and % female) and host biochemical traits (ash, moisture, protein, fiber, fat, carbohydrate, and pH). Pairwise comparisons were performed among five host species with four replicates each (*n* = 20). Pearson’s product–moment correlation analysis was conducted in R. The strength and direction of the correlations are represented by the color intensity (dark red = −1, white = 0, and dark blue = +1).

Based on the correlation matrix presented in Figure 2, several host biochemical traits showed strong associations with pest damage parameters. Percent infestation and percent seed weight loss exhibited very high positive correlations with fat content (r = 0.95 and r = 0.89, respectively) and carbohydrate content (r = 0.78 for both). The number of eggs laid was strongly positively correlated with protein content (r = 0.88) and fat content (r = 0.80). Conversely, fiber content showed moderate to strong negative correlations with number of eggs (r = −0.61) and percent female emergence (r = −0.85), while moisture content was negatively correlated with percent infestation (r = −0.60) and percent adult emergence (r = −0.67). Percent female emergence (second measure) was strongly negatively associated with carbohydrates (r = −0.85) and fiber (r = −0.85). These patterns indicate that host susceptibility is primarily associated with high levels of fats, carbohydrates, and proteins, whereas high fiber and moisture content appear to confer resistance. Therefore, among the evaluated hosts, the one with the highest fat, carbohydrate, and protein content combined with low fiber and low moisture would be the most susceptible to the pest.

*V. unguiculata* was identified as the most susceptible host, as evidenced by maximum infestation and seed weight loss, supported by a biochemical profile characterized by lower structural defenses (fiber) and higher nutritional suitability (carbohydrates and lipids), whereas *P. vulgaris* exhibited strong resistance despite high oviposition, indicating post-oviposition developmental constraints.

It is important to note that the correlation analysis is based on only five host legume species. With such a small sample size, even high correlation coefficients (e.g., r > 0.90) should be interpreted with caution, as they may be influenced by outlier values and do not imply robust predictive or causal relationships. Therefore, the associations reported here are exploratory and hypothesis-generating, rather than confirmatory. Future studies with a larger and more diverse set of host genotypes are needed to validate these patterns.

### 3.4. Biological Responses of C. maculatus to Plant Powder Treatments

**Figure 3:**
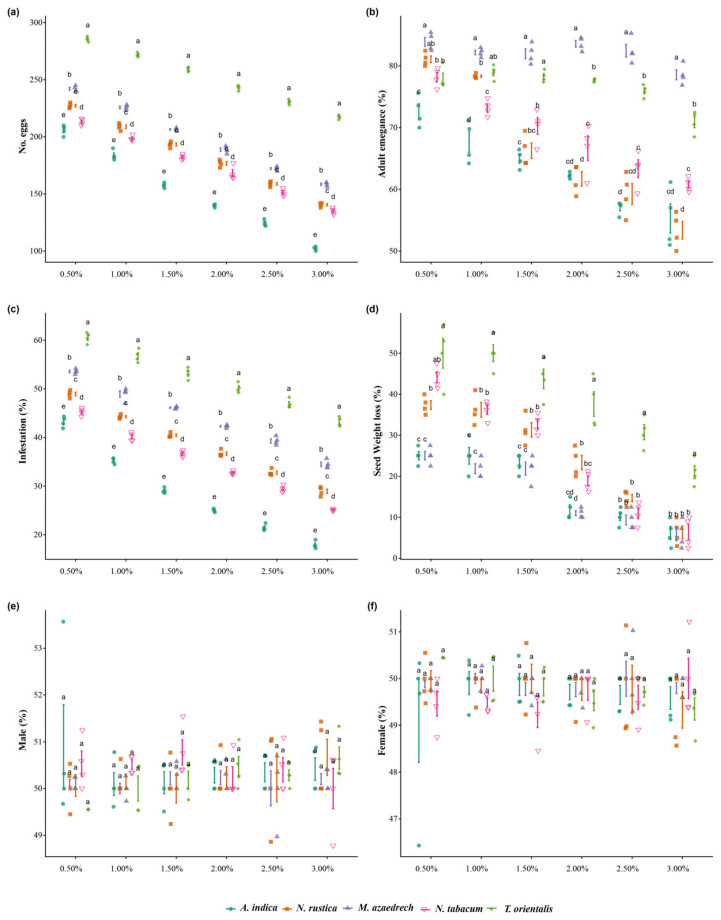
Effect of five botanical powders (*A. indica*, *N. tabacum*, *N. rustica*, *M. azedarach*, and *T. orientalis*) on *C. maculatus* life parameters. (**a**) Number of eggs laid per 50 g seeds, (**b**) mean % adult emergence, (**c**) mean % seed infestation, (**d**) mean % seed weight loss, (**e**) mean % male ratio, and (**f**) mean % female ratio. The *x*-axis for each panel represents six different concentrations (0.50, 1.00, 1.50, 2.00, 2.50, and 3.00% *w*/*v*). Different lowercase letters within each concentration and for each parameter indicate significant differences among plant species at *p* = 0.05 using Tukey’s HSD post hoc test.

The biology of *C. maculatus* was significantly influenced by the application of plant powders, with effects generally increasing as the concentration of the plant treatment increased (Figure 3). A significant decline in oviposition was achieved with all plant species at all concentrations (F = 3089.99, *p* < 0.001). Across the different concentrations, there was a significant interaction between concentration and plant species (F = 14.72, *p* < 0.001), demonstrating that the degree of suppression varied depending on the plant used. Also, in all treatments, adult beetle emergence showed a consistent decline with increasing concentration. Plant species (F = 445.19, *p* < 0.001) and concentration (F = 188.33, *p* < 0.001), which had a notable interaction effect (F = 20.94, *p* < 0.001), affected this response, revealing changes in inhibitory efficiency among plant species. The same pattern was exhibited by seed infestation, which decreased significantly in a concentration-dependent manner. Both concentration (F = 2426.13, *p* < 0.001) and plant species (F = 4065.06, *p* < 0.001) had strong significant effect, while their interaction (F = 14.06, *p* < 0.001) confirmed differences in effectiveness across treatments. Also, host seed weight loss decreased as concentration increased, with significant contributions from plant species (F = 190.20, *p* < 0.001) and concentration (F = 224.78, *p* < 0.001). Among plant species, the interaction effect (F = 4.46, *p* < 0.001) further revealed inconsistency in protective efficiency. In contrast, the proportion of male and female adult emergence remained unaffected by plant species, concentration, or their interaction. For males, plant species (F = 0.99, *p* = 0.4189), concentration (F = 0.35, *p* = 0.8822), and their interaction (F = 1.01, *p* = 0.4551) were not significant, and the same pattern was observed for females, with plant species (F = 0.99, *p* = 0.4184), concentration (F = 0.35, *p* = 0.8813), and interaction (F = 1.01, *p* = 0.4548) all showing no significant effects Tables S7–S12).

### 3.5. Biological Effects of Aqueous Plant Extracts on C. maculatus (F.)

**Figure 4:**
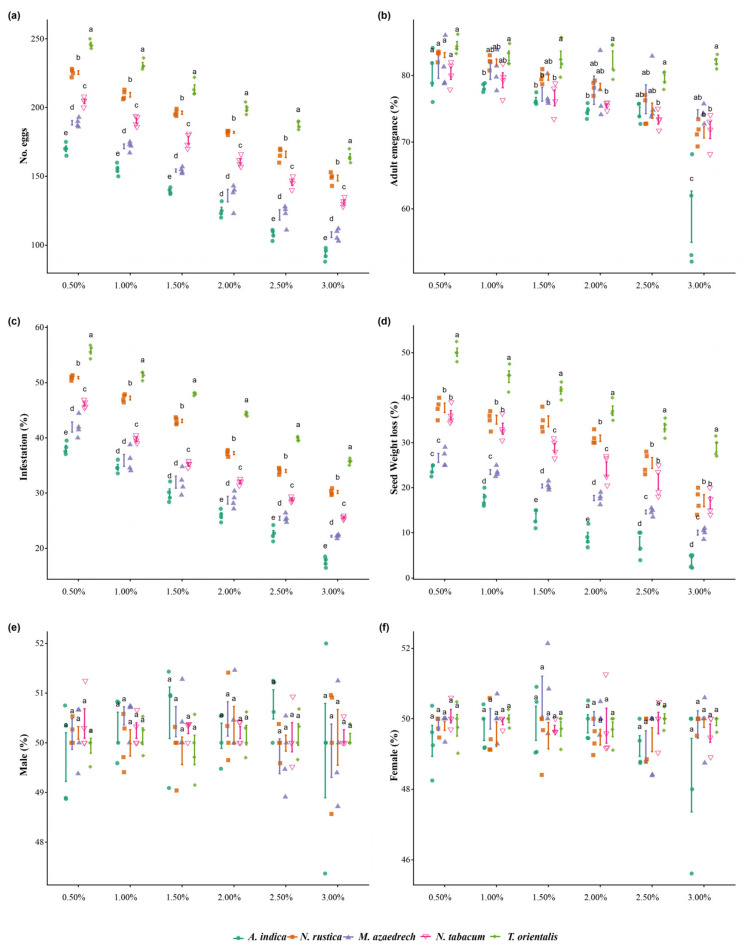
Effect of five aqueous plant extracts (*A. indica*, *N. tabacum*, *N. rustica*, *M. azedarach*, and *T. orientalis*) on *C. maculatus* life parameters. (**a**) Number of eggs laid per 50 g seeds, (**b**) mean % adult emergence, (**c**) mean % seed infestation, (**d**) mean % seed weight loss, (**e**) mean % male ratio, and (**f**) mean % female ratio. The *x*-axis for each panel represents six different concentrations (0.50, 1.00, 1.50, 2.00, 2.50, and 3.00% *w*/*v*). Different lowercase letters within each concentration and for each parameter indicate significant differences among plant species at *p* = 0.05 using Tukey’s HSD post hoc test.

The application of aqueous plant extracts significantly affected the biology of *C. maculatus* (F.), with most responses showing a clear concentration-dependent trend. As the extract concentration increased, oviposition decreased, and both plant species (F = 1330.64, *p* < 0.001) and concentration (F = 1011.84, *p* < 0.001) had highly significant effects. Importantly, across all plant species, the interaction between plant species and concentration was not significant (F = 0.95, *p* = 0.5313), showing a consistent response pattern. Adult emergence also decreased with increasing concentration, with significant effects of plant species (F = 38.52, *p* < 0.001) and concentration (F = 43.99, *p* < 0.001) (Figure 4). In contrast to oviposition, the interaction effect was significant (F = 5.26, *p* < 0.001), showing that the inhibitory effects of plant species varied depending on concentration. Aqueous extracts significantly reduced host seed infestation, with significant effects from both plant species (F = 1276.93, *p* < 0.001) and concentration (F = 1176.57, *p* < 0.001). The interaction was also significant (F = 2.44, *p* = 0.0023), although weaker, showing moderate variation in effectiveness among plant species across concentrations. Similarly, seed weight loss decreased with increasing concentration and was significantly influenced by plant species (F = 630.89, *p* < 0.001) and concentration (F = 250.49, *p* < 0.001). The interaction effect (F = 2.30, *p* = 0.0042) indicated some variation in protective efficiency, though less pronounced than that observed with powder treatments. In contrast, the proportion of male and female adult emergence remained unaffected by plant species, concentration, or their interaction. For males, plant species (F = 0.35, *p* = 0.8420), concentration (F = 0.43, *p* = 0.8277), and interaction (F = 0.64, *p* = 0.8741) were not significant, and for females, plant species (F = 2.27, *p* = 0.0683), concentration (F = 0.98, *p* = 0.4341), and interaction (F = 1.00, *p* = 0.4743) also showed no significant effects (Tables S13–S18).

### 3.6. Concentration-Dependent Biological Effects of Ethanolic Plant Extracts on C. maculatus

**Figure 5:**
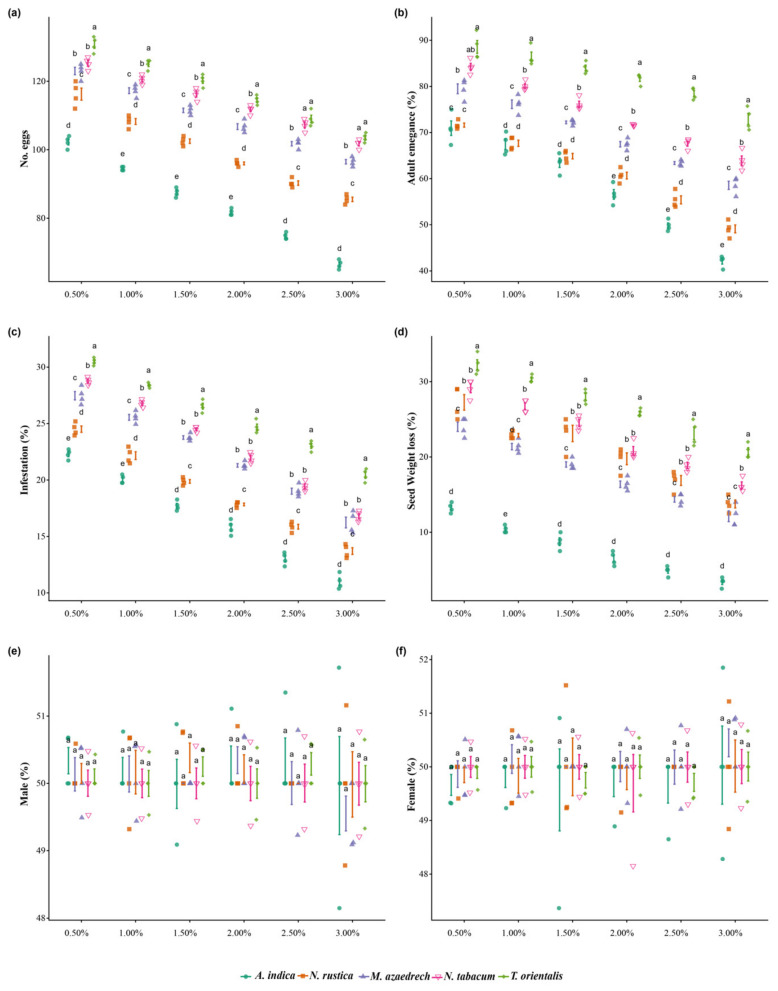
Effect of five ethanolic plant extracts (*A. indica*, *N. tabacum*, *N. rustica*, *M. azedarach*, and *T. orientalis*) on *C. maculatus* life parameters: (**a**) number of eggs laid per 50 g seeds, (**b**) mean % adult emergence, (**c**) mean % seed infestation, (**d**) mean % seed weight loss, (**e**) mean % male ratio, and (**f**) mean % female ratio. The *x*-axis for each panel represents six different concentrations (0.50, 1.00, 1.50, 2.00, 2.50, and 3.00% *w*/*v*). Different lowercase letters within each concentration and for each parameter indicate significant differences among plant species at *p* = 0.05 using Tukey’s HSD post hoc test.

The application of ethanolic plant extracts significantly influenced the biology of *C. maculatus*, with most parameters showing a clear concentration-dependent decline (Figure 5). Oviposition decreased markedly as concentration increased, and both plant species (F = 1895.04, *p* < 0.001) and concentration (F = 1032.02, *p* < 0.001) had highly significant effects. There was also a significant interaction between plant species and concentration significant (F = 5.63, *p* < 0.001). There was species-specific variation in the degree of oviposition, with suppression apparent across all concentrations. Adult emergence followed a similar declining trend, with significant effects of plant species (F = 778.42, *p* < 0.001) and concentration (F = 469.74, *p* < 0.001). A significant interaction was also seen (F = 5.95, *p* < 0.001), revealing that inhibition varied depending on both factors. There was a significant reduction in seed infestation induced by both plant species (F = 1344.56, *p* < 0.001) and concentration (F = 1543.21, *p* < 0.001). The interaction effect was also significant (F = 2.67, *p* = 0.0009), although weaker than that seen for oviposition and adult emergence. This revealed minimum variation in efficacy among the selected plant species across concentrations. Seed weight loss decreased with increasing concentration and was significantly influenced by plant species (F = 984.83, *p* < 0.001) and concentration (F = 318.51, *p* < 0.001). However, the interaction was not significant (F = 1.58, *p* = 0.0758), indicating a consistent reduction pattern across plant species. In contrast, the proportion of male and female adult emergence remained unaffected by plant species, concentration, or their interaction. For males, plant species (F = 0.49, *p* = 0.7428), concentration (F = 0.65, *p* = 0.6649), and interaction (F = 0.30, *p* = 0.9982) were not significant, and for females, plant species (F = 0.83, *p* = 0.5078), concentration (F = 0.48, *p* = 0.7882), and interaction (F = 0.15, *p* = 1.0000) also showed no significant effects (Tables S20–S24).

### 3.7. Toxicity Effect of Plant Powders Against C. maculatus After a 24–96 h Exposure Period

**Figure 6:**
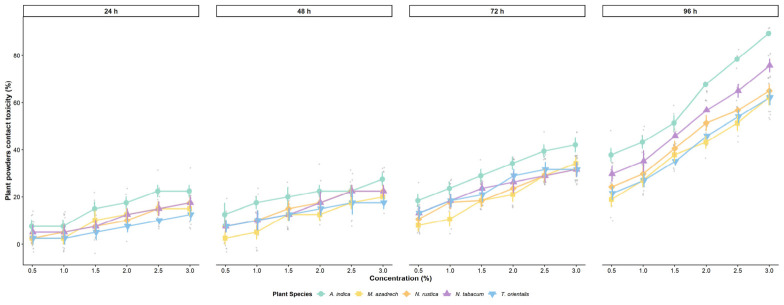
Mean percent mortality (±SE) of *C. maculatus* (F.) after 24 h, 48 h, 72 h, and 96 h of exposure to powders made from five plant species at six different concentrations (0.5–3.0% *w*/*w*). Each treatment combination had five replicate containers (*n* = 5), with 20 adults per replicate. Mortality was corrected using Abbott’s formula. Error bars represent standard error of the mean. Statistical comparisons were made using two-way ANOVA (plant species × concentration) followed by Tukey’s HSD post-hoc test (α = 0.05).

At 24 h, mortality of *C. maculatus* remained relatively low, indicating limited toxicity across treatments. However, both plant species (F = 9.29, *p* < 0.001) and concentration (F = 22.97, *p* < 0.001) significantly influenced mortality (Figure 6). The stronger F-value for concentration suggests that dose-dependent effects dominated early toxicity, while plant-specific differences were present but less pronounced. The plant × concentration interaction was non-significant (*p* = 0.9969), confirming uniform response patterns across species. At this stage, probit analysis indicated relatively high LC_50_ values, showing lower toxicity. *A. indica* exhibited comparatively more potency (LC_50_ = 1.90%), whereas *T. orientalis* showed the lowest toxicity (LC_50_ > 3.00%) among the tested plant species. At this time point, several models showed borderline fits (*p* > 0.05), suggesting unpredictable toxic responses during early exposure (Table S25).

At 48 h, significant effects for both plant species (F = 7.72, *p* < 0.001) and concentration (F = 18.84, *p* < 0.001) on the mortality of *C. maculatus* were observed. The plant-specific differences were more consistent over time. The continued dominance of concentration effects shows that toxicity remained mainly dose-dependent. The interaction was not significant (*p* = 0.9981), supporting the stability of the species response across concentrations. Among all tested plant species, at lethal concentrations, toxicity increased with exposure duration. The least effective toxicity (LC_50_ = 2.50%) was observed for *T. orientalis*, while *A. indica* again showed maximum toxicity (LC_50_ = 1.70%). Probit model fits improved, exhibiting higher consistency in mortality response as compared to 24 h (Table 2 and Table S26).

The effect of both plant species (F = 12.24, *p* < 0.001) and concentration (F = 38.41, *p* < 0.001) become more clear with the 72 h exposure period. The F values increased specifically with concentration, which demonstrates better dose sensitivity with prolonged exposure. Among the tested plant species, the interaction effect remained non-significant (*p* = 0.9931), confirming consistent performance ranking. LC_50_ values continued to decrease, showing higher toxicity. The lowest LC_50_ value (1.40%) was recorded for *A. indica*, followed by *N. tabacum* (1.60%) and *N. rustica* (1.70%), while *T. orientalis* remained the least effective (2.00%). The slope values increased, suggesting steeper dose–response relationships and reduced variability among individuals (Table S27).

At 96 h, both plant species (F = 76.96, *p* < 0.001) and concentration (F = 245.52, *p* < 0.001) had highly significant effects, with higher F-values compared to earlier exposure periods. This trend shows that toxicity increased over time and was especially dependent on concentration. The interaction remained non-significant (*p* = 0.5742), showing that relative plant efficacy remained stable even in high toxicity situations. At this stage, the LC_50_ values were lowest, revealing the highest toxicity. The highest potency (LC_50_ = 1.10%) was showed by *A. indica*, followed by *N. tabacum* (1.30%), *N. rustica* (1.40%), *M. azedarach* (1.50%), and *T. orientalis* (1.60%). Higher slope values and improved probit model fits show robust and consistent toxic responses, proposing reduced heterogeneity in population susceptibility with prolonged exposure (Table S28).

### 3.8. Toxic Effects of Plant Aqueous Extracts Against C. maculatus After a 24–96 h Exposure Period

**Figure 7:**
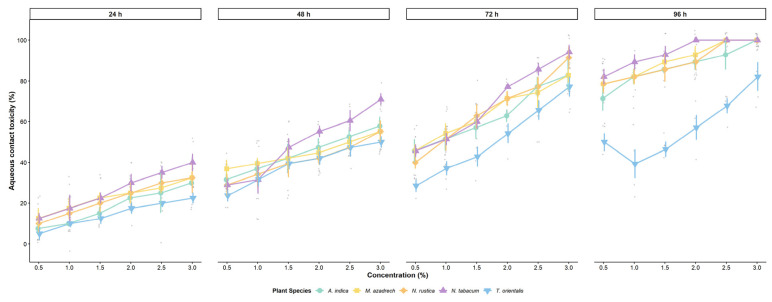
Mean percent mortality (±SE) of *C. maculatus* (F.) after 24 h, 48 h, 72 h, and 96 h of exposure to aqueous extracts of five plant species at six different concentrations (0.5–3.0% *w*/*v*). Each treatment combination had five replicate containers (*n* = 5), with 20 adults per replicate. Mortality was corrected using Abbott’s formula. Error bars represent standard error of the mean. Statistical comparisons were made using two-way ANOVA (plant species × concentration) followed by Tukey’s HSD post hoc test (α = 0.05).

At 24 h, contact toxicity of plant crude extracts to *C. maculatus* was moderate, exhibiting partial early-stage lethality. Across all plant species, the mortality increases gradually, which is also reflected in the figure by the relatively shallow dose–response curves (Figure 7). ANOVA showed that both plant species (F = 10.62, *p* < 0.001) and concentration (F = 23.52, *p* < 0.001) significantly affected mortality (Table S29). The maximum F-value at this early stage for concentration shows that dose-dependent effects dominated toxicity, while plant-specific differences were secondary. The interaction of plant × concentration was non-significant (*p* = 1.0000), revealing consistent response patterns across species. Probit analysis exhibited relatively high LC_50_ values, showing lower toxicity with short exposure times. Among the tested plant species, *A. indica* exhibited the highest toxicity (LC_50_ = 1.60%), whereas *T. orientalis* showed the lowest (LC_50_ = 2.80%) Table 3. Some models showed borderline fit (e.g., *T. orientalis*, *p* ≈ 0.06), suggesting greater variability and instability in early mortality response.

At 48 h, mortality increased significantly, showing progressive toxic effects over time. Dose–response curves became steeper compared to 24 h, reflecting increased sensitivity to concentration. Both plant species (F = 4.92, *p* = 0.0013) and concentration (F = 29.84, *p* < 0.001) significantly influenced mortality. The stronger effect of concentration confirms that toxicity remained primarily dose-driven, although plant effects became more consistent. The interaction remained non-significant (*p* = 0.6059), indicating stable ranking among species. LC_50_ values decreased across all plants, confirming enhanced toxicity with prolonged exposure. *A. indica* (1.30%) remained the most effective, followed by *N. tabacum* (1.70%) and *N. rustica* (1.80%), while *T. orientalis* remained least effective (2.30%) (Table S30). Improved probit fits suggest greater stability in mortality response compared to 24 h.

At 72 h, mortality increased further, indicating significant toxic impact with prolonged exposure. The figure shows steeper and more convergent dose–response curves, reflecting reduced variability among individuals. Both plant species (F = 16.15, *p* < 0.001) and concentration (F = 79.23, *p* < 0.001) exerted highly significant effects. The sharp increase in F-values, particularly for concentration, indicates amplified dose sensitivity at this stage. The interaction remained non-significant (*p* = 0.8699), confirming consistent dose–response relationships across species. LC_50_ values declined further, indicating increased toxicity. *A. indica* showed the lowest LC_50_ (1.00%), followed by *N. tabacum* (1.30%) and *N. rustica* (1.40%), whereas *T. orientalis* remained least effective (1.80%). Increasing slope values suggest steeper dose–response relationships and reduced heterogeneity in susceptibility Table S31).

At 96 h, mortality reached its highest level, indicating near-maximal toxic effects across treatments. Dose–response curves approached saturation, with high mortality even at intermediate concentrations. Both plant species (F = 89.27, *p* < 0.001) and concentration (F = 29.92, *p* < 0.001) remained highly significant. Notably, the plant effect exceeded the concentration effect at this stage, suggesting that species-specific biochemical properties became more influential with prolonged exposure. The interaction remained non-significant (*p* = 0.0798), indicating stable response patterns. LC_50_ values were lowest at 96 h, confirming maximum toxicity. *A. indica* showed the highest potency (0.80%), followed by *N. tabacum* (0.90%) and *N. rustica* (1.00%), while *T. orientalis* remained least effective (1.40%). Probit models showed strong fit and higher slope values, indicating consistent and uniform toxic responses (Table S32).

### 3.9. Toxic Effects of Plant Ethanolic Extracts Against C. maculatus After a 24–96 h Exposure Period

**Figure 8:**
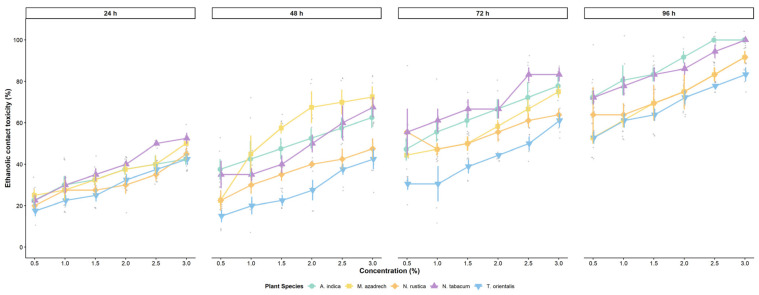
Mean percent mortality (±SE) of *C. maculatus* (F.) after 24 h, 48 h, 72 h, and 96 h of exposure to ethanolic extracts of five plant species at six different concentrations (0.5–3.0% *w*/*v*). Each treatment combination had five replicate containers (*n* = 5), with 20 adults per replicate. Mortality was corrected using Abbott’s formula. Error bars represent standard error of the mean. Statistical comparisons were made using two-way ANOVA (plant species × concentration) followed by Tukey’s HSD post hoc test (α = 0.05).

At 24 h, mortality increased slowly with concentration, as reflected by the relatively shallow dose–response patterns in Figure 8. ANOVA showed that mortality was significantly affected by both plant species (F = 13.10, *p* < 0.001) and concentration (F = 18.04, *p* < 0.001) (Table S33). However, across plant species, the non-significant plant × concentration interaction (*p* = 0.9777) shows similar response trends. Probit analysis revealed comparatively high LC_50_ values, confirming low early toxicity. Among the tested plants, *A. indica* showed the highest efficacy (LC_50_ = 1.20%), while *T. orientalis* showed the lowest (LC_50_ = 2.40%) (Table 4). Model fit was acceptable but less robust than at later time points, showing greater variability in early-stage mortality.

Dose–response curves became more defined, showing increased sensitivity to concentration at 48 h. Both plant species (F = 10.29, *p* < 0.001) and concentration (F = 43.41, *p* < 0.001) significantly influenced mortality, with concentration exerting a stronger effect. The interaction remained non-significant (*p* = 0.9990), confirming stable dose–response relationships across plant species (Table S34). LC_50_ values decreased across all species, indicating enhanced toxicity. *A. indica* remained the most effective (LC_50_ = 1.00%), followed by *N. tabacum* (1.30%) and *N. rustica* (1.40%), whereas *T. orientalis* remained least effective (1.90%) (Figure 8). Improved probit fits suggest greater consistency compared to 24 h.

The mortality increased significantly with 72 h of exposure, suggesting intensified toxic effects with prolonged exposure. In Figure 8, the dose–response curves became steeper, reflecting declining variability and stronger concentration dependence. There is a highly significant effect for both plant species (F = 18.18, *p* < 0.001) and concentration (F = 67.86, *p* < 0.001) (Table S35). The continued dominance of concentration shows strong dose-dependent toxicity, while plant effects became more obvious. The interaction remained non-significant (*p* = 0.9999). LC_50_ values declined further, with *A. indica* showing the most potency (0.80%), followed by *N. tabacum* (1.00%) and *N. rustica* (1.10%). *T. orientalis* remained least effective (1.50%). Increasing slope values show steeper dose–response relationships and reduced heterogeneity in susceptibility.

At 96 h of exposure, the mortality of *C. maculatus* reached a high level, confirming strong cumulative toxicity. Dose–response curves approached saturation at higher concentrations, especially for the more effective plant species. Both plant species (F = 62.97, *p* < 0.001) and concentration (F = 128.63, *p* < 0.001) exhibited the most significant effects at this exposure duration (Table S36). Unlike the aqueous contact toxicity dataset, concentration remained dominant even at this stage. This confirms that dose continues to govern ethanolic toxicity with extended exposure. Again, the interaction is non-significant (*p* = 0.9996). At 96 h of exposure, the LC_50_ values were lowest, indicating maximum toxicity. Highest efficacy (0.60%) was shown by *A. indica*, followed by *N. tabacum* (0.80%) and *N. rustica* (0.90%), while *T. orientalis* remained the least effective (1.20%) (Figure 8). Probit models revealed strong fit and high slope values, showing consistent and predictable mortality responses.

Because extraction yields and marker-compound concentrations were not standardized, the reported efficacy rankings among plant species and between solvents should be interpreted with caution. Differences in activity may reflect variation in extraction efficiency, as well as intrinsic bioactivity.

It is important to note that aqueous extracts were tested using contact toxicity, whereas ethanolic extracts and powders were tested using residual toxicity (see Section 2.8). Therefore, direct comparisons of LC_50_ values between aqueous extracts and the other two formulations should be made cautiously, as the exposure routes differ.

## 4. Discussion

The present study provides an integrated analysis of the biochemical and toxicological determinants of *C. maculatus* (F.) infestation across five legume host seeds. It also showed a systematic evaluation of the biological and insecticidal activities of plant-based powders, aqueous extracts, and ethanolic extracts from five different plant species. There were three major finding: (i) an obvious decoupling between oviposition preference and progeny performance, driven by species-specific biochemical profiles; (ii) a consistent hierarchy of host susceptibility, where *V. unguiculata* was most vulnerable and *P. vulgaris* (L.) exhibited high resistance despite maximum oviposition, and (iii) concentration- and time-dependent toxicity of plant-based treatments, with *A. indica* and *N. tabacum* (L.) exhibiting the highest efficacy, especially when extracted with ethanol. These results have significant implications for understanding host–pest coevolution and for developing evidence-based, economically feasible, socially acceptable, and easily available product protection strategies.

### 4.1. Host Preference and the Oviposition–Performance Paradox

The free-choice oviposition assay showed that *C. maculatus* females laid the highest number of eggs on *P. vulgaris* (166.80 ± 1.34), but this host exhibited the lowest adult emergence (4.00 ± 0.33%), infestation rate (3.00 ± 0.59%), and seed weight loss (2.00 ± 0.31%). On the other hand, oviposition was lower on *V. unguiculata* (L.), yet this host showed the highest seed weight loss (53.00 ± 0.94%), and near-maximal adult emergence was observed on *V. radiata* L. R. Wilczek (96.00 ± 0.46%). This obvious oviposition performance mismatch is a well-documented phenomenon in bruchid beetles and is often described as an evolutionary trap or host deception [22]. *C. maculatus* females select host seeds mostly based on surface cues (e.g., texture, seed size, volatile allelochemicals), which may not reliably show internal nutritional quality or the presence of toxic secondary metabolites [9,44]. *P. vulgaris* showed strong post-ovipositional resistance (antibiosis) that is not perceived by ovipositing females, whereas *V. unguiculata* may provide a more suitable environment for larval development due to comparatively lower defensive constraints.

Our free-choice assay standardized host seed mass (50 g) rather than seed number or surface area. Legume species differ substantially in seed size: for example, 50 g of *P. vulgaris* contains many more individual seeds than 50 g of *V. unguiculata* or *C. arietinum* L. Consequently, the higher total number of eggs recorded on *P. vulgaris* could be partly due to greater available oviposition surface area or a higher number of seed units, rather than solely reflecting a behavioral preference for that species. Therefore, while the mismatch between egg distribution and adult emergence is robust (i.e., many eggs on *P. vulgaris* but very low emergence), the magnitude of the difference in oviposition among hosts should not be interpreted as a pure measure of female choice. Future studies should standardize by seed number or surface area to disentangle morphological and behavioral effects.

The biochemical profiling (Section 3.2) and correlation analysis (Section 3.3) deliver an explanation for this paradox. *P. vulgaris* showed maximum fiber content (z = 1.25) and clustered with *C. arietinum* in a group characterized by higher ash and fiber content. Fiber content was negatively correlated with number of eggs (r = −0.61) and more strongly correlated with the percentage of female emergence (r = −0.85). Larval penetration and feeding may be hindered by higher fiber content, which likely acts as a physical barrier. It may also relate to cell wall-bound phenolic compounds that reduce digestion [45,46]. Fiber content showed a strong negative relationship with adult emergence (r = −0.85), suggesting that higher fiber levels reduced insect survival. Fiber may act as a physical barrier to feeding and development. Although this study did not measure phenolic or enzyme inhibitors, previous studies have reported that common beans contain α-amylase inhibitors that can increase larval mortality [47,48]. Our correlational data are consistent with this possibility, but direct evidence from our measurements is lacking. These factors may explain why oviposition on *P. vulgaris* seeds does not translate into adult emergence, as eggs hatch, but the neonate larvae fail to use the endosperm. On the other hand, *V. unguiculata* showed higher carbohydrate (z = 1.33) and reasonable fat levels with comparatively low fiber (z = −0.60). This created an easily accessible, energy-dense resource that increases larval survival.

Further, the correlation matrix shows a strong relationship between infestation parameters and seed composition. Percent infestation and % seed weight loss were both highly correlated with fat content (r = 0.95 and 0.89, respectively), as well as with carbohydrate content (r = 0.78 for both). Proteins content was highly associated with egg number (r = 0.88). These findings corroborate earlier reports that macronutrient availability, particularly amino acid reserves, supports vitellogenesis in *C. maculatus* [49,50]. Taken together, these findings suggest that a hierarchy of susceptibility emerges from a trade-off between nutritional benefits and structural or defensive constraints. Nutritional benefits include fats, carbohydrates, and proteins, whereas the constraints include fiber, moisture, and specific antinutrients. *V. unguiculata* sits at the susceptible extreme and *P. vulgaris* at the resistant extreme, and other hosts occupy intermediate positions.

### 4.2. Biochemical Divergence Among Legume Hosts

Hierarchical clustering of the seven nutritional parameters revealed two major groups: (i) *P. sativum* L., *V. radiata*, and *V. unguiculata* and (ii). *P. vulgaris* with *C. arietinum* L. This separation appeared to be determined by differences in fiber, ash, and moisture contents. The close clustering of chickpea and common beans is notable because both are known to contain lectins and enzyme inhibitors that confer resistance to *C. maculatus* [51,52]. *P. sativum* was characterized by relatively high fat (z = 1.55) and protein (z = 1.41) contents, but it did not support a maximum level of infestation or seed weight loss. This pattern may be associated with the presence of deterrent compounds such as saponins and with structural traits such as seed coat hardness [53]. Due to high moisture content (z = 1.66), *V. radiata* showed low % infestation (r = −0.60) and % adult emergence (r = −0.67) (not statistically robust given *n* = 5). Moisture may have two effects: physiological (less moisture minimizes fungal growth, but also affects beetle water balance) and physical (dry seeds are harder and less feasible for larval tunneling). Overall, the multivariate approach clearly revealed that no single trait determines susceptibility; rather, it is the mixture of several nutrients and defenses that is associated with observed results. However, the clustering and correlation analyses are based on only five host species; therefore, these patterns should be considered exploratory and hypothesis-generating rather than confirmatory. The fact that pH was only associated with moderate changes and did not drive cluster separation corroborates earlier findings. Bruchids can tolerate a varied range of gut pH values [54]. Therefore, pH is unlikely to be a main factor in host selection or larval performance in this system.

### 4.3. Botanical Treatments: Concentration, Time, and Solvent Effects

The second part of the study evaluated plant-based insecticides in three formulations (powder, aqueous extract, ethanolic extract) derived from five different plant species against *C. maculatus*, focusing on biological performance and mortality. Many consistent patterns emerged across all assays.

#### 4.3.1. Concentration-Dependent Suppression of Biological Parameters

There was significant reduction in oviposition, % adult emergence, % seed infestation, and % seed weight loss as the concentration of plant species treatment increased. This dose–response relationship is the symbol of effective plant-based insecticides and has been extensively reported for neem, tobacco, and other plant species [55,56]. Maximum F-values for concentration (e.g., for oviposition with powder treatments: F = 3089.99, *p* < 0.001) shows that *C. maculatus* biological parameters are highly sensitive to increasing concentrations of active compounds. Notably, the plant species × concentration interaction was non-significant or small in magnitude in most cases. This suggests that, across plant species, the slopes of the dose–response curves were similar. Across the tested plant species, this indicates that the mechanism of action, i.e., antecedency, oviposition deterrence, or toxicity, is qualitatively similar. However, their absolute potencies differ.

#### 4.3.2. Extraction Solvent Determines Efficacy and Consistency

There was clear chain of potency, with ethanolic extracts exhibiting the highest activity, followed by aqueous extracts and then powders. However, direct comparisons between contact-based (aqueous) and residual-based (ethanolic, powder) toxicity are not possible, because the exposure routes differ. The observed hierarchy should therefore be interpreted as a general trend consistent with the higher bioavailability of ethanolic extracts, rather than as a definitive potency ranking across formulations. This hierarchy was based on the lowest LC_50_ values and the steeper dose–response curves. For example, at 96 h of exposure, the *A. indica* A. Juss. ethanolic extract had an LC_50_ of 0.60%, whereas the aqueous extract of the same plant had an LC_50_ of 0.80%, and the plant powder required 1.10% to achieve 50% mortality. This pattern is expected, because several bioactive molecules of insecticidal plants, including azadirachtin (from *A. indica*) and nicotine (from *Nicotiana* spp.), are non-polar, and ethanol is a far more effective solvent for extracting such compounds. Alkaloids and other limonoids are water soluble [14,57]. Ethanolic extraction thus releases a maximum concentration of active compounds, resulting in greater lethality even at lower doses. Also, ethanolic extracts showed more consistent probit model fits (lower heterogeneity) and steeper slopes, indicating a more uniform and predictable toxic response. This has practical implications: while powders are cheapest and easiest to prepare, ethanolic extracts are more suitable for standardized, high-efficacy applications.

#### 4.3.3. Temporal Progression of Toxicity

For all three formulations, *C. maculatus* mortality significantly increased from 24 h to 96 h. For the aqueous extracts, the mortality of *C. maculatus* increased from 26.33% at 24 h to 83.61% at 96 h; for the ethanolic extracts, from 9.00% to 60.63%; and for the powders, from 10.33% to 47.68%. This time-dependent increase reflect a delayed or accumulative toxic effect of plant-derived bioactive compounds. For example, azadirachtin disrupts ecdysteroid synthesis and inhibits feeding behavior, with lethality often taking several days after exposure [58,59]. Nicotine acts as a neurotoxin by targeting nicotinic acetylcholine receptors, but its effectiveness depends on sufficient penetration and systemic exposure. The lack of a significant plant × concentration interaction shows that the ranking of plant species remained stable over time. Among the tested plant species, *A. indica* consistently exhibited the highest efficacy, followed by *N. tabacum* L., *M. azedarach* L., and *T. orientalis* L., which encourages its selection as a particularly effective candidate for practical application.

At 96 h of exposure, a notable difference between aqueous extracts and ethanolic extracts was seen. For the aqueous extracts, the effect of plant species (F = 89.27) exceeded that of concentration (F = 29.92), whereas for the ethanolic extracts, the concentration effect (F = 128.63) remained dominant. This revealed that, with prolonged contact, the specific chemical composition of each plant species becomes increasingly important. For example, neem may show higher activity due to the synergistic effects of multiple limonoids, while with ethanolic treatments, the high initial concentration of active compounds continues to drive mortality, regardless of minor inter-species differences.

### 4.4. Comparative Efficacy of Plant Species

Across all assays and exposure periods, the most consistently effective plant species was *A. indica*, followed by *N. tabacum* and *N. rustica* L., then *M. azedarach*, and finally *T. orientalis* as the least effective. In stored-product entomology, the dominance of *A. indica* is well-established; azadirachtin and other tetranortriterpenoids are known to exert potent antifeedant, growth-regulating, and oviposition-deterrent effects at low concentrations [60,61]. Here, the LC_50_ values obtained for *A. indica* powder (1.10% after 96 h) and *A. indica* ethanolic extract (0.60% after 96 h) are comparable to or better than many literature reports [40,62]. *Nicotiana* species exhibited the second-highest efficacy, due to the presence of nicotine, a quick-acting neurotoxin, as well as other alkaloids such as nornicotine and anabasine that may exert additive effects [63,64]. *M. azedarach* showed reasonable efficacy consistent with its limonoid content (meliantriol, toosendanin), which is generally less potent than azadirachtin but still showed bioactivity [65]. The least effective plant species is *T. orientalis*, which was consistently poor; its main constituents (thujone, camphor) are volatile and may not persist or penetrate effectively in a seed-treatment context [66]. Importantly, the ethanolic extract of *T. orientalis* still showed some toxicity, with an LC_50_ of 1.20% at 96 h, but it was always the weakest. Nevertheless, these toxicity comparisons should be interpreted cautiously, because LC_50_ estimates are accompanied by confidence intervals that vary among treatments and exposure periods. Furthermore, goodness-of-fit statistics indicated differences in model precision among datasets. Therefore, the efficacy rankings reported in this study should be considered relative trends in toxicity rather than definitive evidence of statistically distinct potency among all botanical treatments.

### 4.5. Resolving the Apparent Paradox Between Oviposition and Adult Toxicity

The highest oviposition, infection, and weight loss (Figure 3, Figure 4 and Figure 5), yet the lowest LC_50_ (highest adult mortality) in direct toxicity assays (Table 2, Table 3 and Table 4, Figure 6, Figure 7 and Figure 8), observed with *A. indica* treatments gives rise to an apparent paradox. A rational solution for this incongruence takes into account various routes of exposure, as well as the duration of exposure. In the oviposition assay, the females laid eggs within 24 to 48 h of exposure. This time window is too short to be affected by the delayed action of azadirectin (ecdysteroid disruption, antifeedancy) to cause adult mortality and completely deter the oviposition [57,58,59]. After hatching, the larvae come into contact with limonoids in the seed matrix, and seed damage occurs prior to larval death, which is typical of botanical insecticides with chronic toxicity [55,60]. Adult mortality assays, on the other hand, measure the direct acute toxicity (24–96 h) without seed residue, where adult escape is highly unlikely, thus allowing rapid mortality. Hence, the two datasets are not contradictory, but complementary. *A. indica* exhibits a high level of direct acute lethal toxicity in adults; however, it is not fully effective against early oviposition in the tested seed treatments.

### 4.6. Implications for Integrated Pest Management (IPM) of Stored Pulses

The combined results provide a clear, evidence-based framework for managing stored *C. maculatus* (F.). First, host selection at the storage stage may serve as a preventive strategy. Where possible, farmers and storekeepers should prioritize *P. vulgaris* or *C. arietinum* over *V. unguiculata* or *V. radiata*, because the former two exhibit natural resistance that reduces adult emergence by >95% despite egg deposition. However, cowpea and mung bean are nutritionally and economically important in many regions, requiring alternative protection. Second, for susceptible hosts, botanical treatments were effective. *A. indica* powder at 1–2% (*w*/*w*) reduced infestation and seed damage substantially, although this inference is based on observed mortality trends rather than direct protection estimates at higher concentrations. Neem leaf powder is a readily accessible option for smallholder farmers [67]. For larger-scale or commercial storage, ethanolic extracts provide superior potency and consistency. The fact that ethanolic neem extract had an LC_50_ of 0.60% after 96 h means that only 6 g of extract per kg of seed could kill half the weevil population; a 2–3% concentration would likely give near-complete control. Third, the temporal dynamics show that treatments should be applied at least 72–96 h before expected infestation, or that stored seeds should be treated immediately after harvest to allow the toxic effect to accumulate before oviposition peaks.

A further advantage of the tested botanicals is that they are generally recognized as safe (GRAS) for use on food commodities, especially when applied as powders or water extracts [68,69,70,71]. Neem and tobacco have been used traditionally for centuries, with minimal mammalian toxicity when applied topically to seeds (except that appropriate protective measures would be needed for nicotine’s high acute toxicity). The lack of effect on sex ratio also means that these treatments do not create unexpected demographic rebounds or selection for resistant alleles that target sex determination pathways.

### 4.7. Limitations

It is important to recognize reservations regarding the present study concerned with mechanistic approach, as the biochemical analysis focused on direct measurement of moisture, ash, macronutrients, fiber, and pH. Indeed we did not measure secondary metabolites like alkaloids, tannins, phenolics, enzyme inhibitors like protease inhibitors, α-amylase inhibitors, and physical parameters like hardness and thickness of seed coat, which could yield definitive rather than correlational interpretations of associations between seed traits and insect performance. The specific resistance mechanisms of α-amylase inhibitors in *P. vulgaris* should be investigated using actual study data, not discussed on the basis of previous literature. Further studies should focus on actual measurements of all the physical and chemical parameters to obtain consequential results.

The lack of characterization of botanical extracts is another gap in the present research work, as we have reported all the treatments as being crude based on original plant material (for example, percentage concentrations like *w*/*w* for powders and *w*/*v* for extracts). We were not able to determine the dry extract recovery, i.e., extraction yields, and did not quantify the marker compounds such as nicotine in *Nicotiana* spp. or azadirachtin in *A. indica*. This gap means that our comparisons of efficacy among different plant species and between types of solvent were based on crude material other than equivalent chemical composition. For instance, a 1% of extract of *A. indica* in ethanol has a different absolute concentration of bioactive compounds as compared to a 1% of ethanolic extract of *T. orientalis*. So, the comparative grading of *A. indica* > *N. tabacum* > others is quite preliminary. This can be improved by using HPLC or GC-MS quantification of all the known marker compounds and finding extraction yields to have standardized comparisons and reproducibility of results.

## 5. Conclusions

The findings of this study indicate a correlation between nutritional macronutrients (fat, carbohydrate, and protein) and defensive components (fiber and moisture) and host susceptibility to *C. maculatus* (F.). This analysis however, is only exploratory, since it was carried out with five host species. For further elaboration and confirmation, a larger and wider host set need to be explored. Moreover, *V. unguiculata* exhibited the highest susceptibility, whereas maximum resistance was demonstrated by *P. vulgaris L*. With the current dataset, the exact mechanism involved could not be established. However, the existing literature and the inverse relationship between adult emergence and the amount of fiber in the plant indicate that physical barriers and possible antinutritive compounds (such as enzyme inhibitors) may have a role in the development of resistance in *P. vulgaris*. Direct measurements of these traits are needed to confirm causality. Botanical treatments, especially ethanolic extracts of *A. indica* A. Juss, caused concentration- and time-dependent reductions in oviposition, % adult emergence, % infestation, % host seed weight loss, and direct mortality, without affecting sex ratio. Strong dose–response relationships and low LC_50_ values (down to 0.60%) recommend neem-based formulations as practical, accessible, and effective tools for integrated management of cowpea weevils in stored pulses. Future work should focus on field validation, compound level characterization, and formulation stability to bridge the gap between laboratory efficacy and on-farm adoption.

## Figures and Tables

**Table 1:** Biology of *C. maculatus* (F.) on different hosts in the free-choice test.

Plant Species	No. of Eggs	Adult Emergence (%)	Infestation (%)	Weight Loss (%)	Sex Ratio	
					Female (%)	Male (%)
*P. vulgaris* L.	166.80 ± 1.34 a	4.00 ± 0.33 e	3.00 ± 0.59 d	2.00 ± 0.31 c	50.40 ± 2.60 a	49.60 ± 2.60 a
*C. arietinum* L.	100.00 ± 0.82 d	67.00 ± 1.24 c	62.00 ± 1.78 a	36.00 ± 1.20 b	49.39 ± 0.40 a	50.61 ± 0.40 a
*P. sativum* L.	88.30 ± 1.34 e	47.00 ± 2.27 d	44.00 ± 1.20 c	33.00 ± 1.39 b	50.36 ± 0.36 a	50.24 ± 0.79 a
*V. radiata* L. R. Wilczek	134.00 ± 1.78 c	96.00 ± 0.46 a	52.00 ± 0.53 b	48.00 ± 0.60 a	49.43 ± 0.24 a	50.38 ± 0.26 a
*V. unguiculata* (L.) Walp.	141.80 ± 1.34 b	86.00 ± 0.39 b	69.00 ± 1.43 a	53.00 ± 0.94 a	49.80 ± 0.27 a	50.20 ± 0.27 a
LSD	8.6054	7.2301	7.2851	5.8348	3.6142	3.7396
F–value	124	232	115	109	0.16	0.09
*p*-value	0.0000	0.0000	0.0000	0.0001	0.9534	0.9841

Means in columns followed by different letters are significantly different at a 5% level of significance (LSD test). Values are presented as mean ± standard error (SE).

**Table 2:** Probit regression analysis of plant powder extracts across exposure periods (LC_50_ estimation).

Species	Time (h)	df	Regression Equation (Probit)	Slope ± SE	LC_50_ (%) (95% CI)	χ^2^	*p*-Value
*A. indica* A. Juss.	24	4	Y = 1.12 + 0.98X	1.22 ± 0.18	1.90 (1.65–2.18)	2.31	0.041
*A. indica* A. Juss.	48	4	Y = 1.05 + 1.10X	1.35 ± 0.16	1.70 (1.48–1.95)	2.67	0.035
*A. indica* A. Juss.	72	4	Y = 0.98 + 1.25X	1.53 ± 0.14	1.40 (1.22–1.62)	3.02	0.022
*A. indica* A. Juss.	96	4	Y = 0.85 + 1.45X	1.78 ± 0.12	1.10 (0.95–1.28)	3.55	0.015
*N. rustica* L.,	24	4	Y = 1.30 + 0.90X	1.10 ± 0.20	2.40 (2.10–2.78)	2.12	0.055
*N. rustica* L.,	48	4	Y = 1.20 + 1.05X	1.30 ± 0.17	2.10 (1.85–2.42)	2.54	0.042
*N. rustica* L.,	72	4	Y = 1.05 + 1.20X	1.50 ± 0.14	1.70 (1.48–1.95)	3.10	0.021
*N. rustica* L.,	96	4	Y = 0.95 + 1.35X	1.62 ± 0.13	1.40 (1.20–1.63)	3.44	0.031
*M. azedarach* L.,	24	4	Y = 1.35 + 0.85X	0.98 ± 0.21	2.60 (2.25–3.00)	2.45	0.060
*M. azedarach* L.,	48	4	Y = 1.20 + 1.00X	1.20 ± 0.18	2.30 (2.00–2.65)	2.60	0.048
*M. azedarach* L.,	72	4	Y = 1.05 + 1.15X	1.40 ± 0.15	1.90 (1.65–2.20)	3.05	0.033
*M. azedarach* L.,	96	4	Y = 0.90 + 1.30X	1.50 ± 0.14	1.50 (1.30–1.75)	3.12	0.025
*N. tabacum* L.,	24	4	Y = 1.25 + 0.95X	1.18 ± 0.19	2.30 (2.00–2.65)	2.29	0.045
*N. tabacum* L.,	48	4	Y = 1.10 + 1.08X	1.32 ± 0.16	2.00 (1.75–2.30)	2.60	0.38
*N. tabacum* L.,	72	4	Y = 0.95 + 1.28X	1.55 ± 0.14	1.60 (1.40–1.85)	3.20	0.024
*N. tabacum* L.,	96	4	Y = 0.80 + 1.40X	1.65 ± 0.13	1.30 (1.12–1.52)	3.50	0.017
*T. orientalis* L.,	24	4	Y = 1.40 + 0.80X	0.85 ± 0.22	>3.00 (2.60–>3.0)	2.60	0.070
*T. orientalis* L.,	48	4	Y = 1.25 + 0.95X	1.10 ± 0.19	2.50 (2.20–2.90)	2.75	0.052
*T. orientalis* L.,	72	4	Y = 1.10 + 1.10X	1.35 ± 0.15	2.00 (1.75–2.30)	3.00	0.041
*T. orientalis* L.,	96	4	Y = 0.95 + 1.20X	1.45 ± 0.14	1.60 (1.40–1.85)	3.08	0.028

**Table 3:** Probit regression analysis of aqueous plant extracts (24–96 h exposure).

Species	Time (h)	df	Regression Equation (Probit)	Slope ± SE	LC_50_ (%) (95% CI)	χ^2^	*p*-Value
*A. indica* A. Juss.	24	4	Y = 1.20 + 1.05X	1.55 ± 0.15	1.60 (1.40–1.85)	2.88	0.030
*A. indica* A. Juss.	48	4	Y = 1.05 + 1.20X	1.80 ± 0.13	1.30 (1.15–1.50)	3.20	0.020
*A. indica* A. Juss.	72	4	Y = 0.90 + 1.35X	2.05 ± 0.11	1.00 (0.88–1.18)	3.50	0.012
*A. indica* A. Juss.	96	4	Y = 0.75 + 1.50X	2.20 ± 0.10	0.80 (0.70–0.95)	3.60	0.008
*N. rustica* L.,	24	4	Y = 1.35 + 0.95X	1.30 ± 0.17	2.20 (1.95–2.55)	2.60	0.045
*N. rustica* L.,	48	4	Y = 1.20 + 1.10X	1.60 ± 0.15	1.80 (1.60–2.10)	2.95	0.033
*N. rustica* L.,	72	4	Y = 1.05 + 1.25X	1.85 ± 0.12	1.40 (1.20–1.65)	3.25	0.020
*N. rustica* L.,	96	4	Y = 0.90 + 1.40X	2.05 ± 0.11	1.00 (0.85–1.20)	3.45	0.014
*M. azedarach* L.,	24	4	Y = 1.40 + 0.85X	1.10 ± 0.18	2.60 (2.30–3.05)	2.50	0.050
*M. azedarach* L.,	48	4	Y = 1.25 + 1.00X	1.40 ± 0.15	2.20 (1.95–2.55)	2.85	0.038
*M. azedarach* L.,	72	4	Y = 1.10 + 1.20X	1.75 ± 0.13	1.70 (1.50–2.00)	3.10	0.025
*M. azedarach* L.,	96	4	Y = 0.95 + 1.35X	1.95 ± 0.12	1.20 (1.05–1.45)	3.30	0.016
*N. tabacum* L.,	24	4	Y = 1.30 + 1.00X	1.45 ± 0.16	2.10 (1.85–2.45)	2.75	0.028
*N. tabacum* L.,	48	4	Y = 1.15 + 1.15X	1.75 ± 0.13	1.70 (1.50–2.00)	3.05	0.018
*N. tabacum* L.,	72	4	Y = 1.00 + 1.30X	2.00 ± 0.11	1.30 (1.10–1.55)	3.35	0.010
*N. tabacum* L.,	96	4	Y = 0.85 + 1.45X	2.20 ± 0.10	0.90 (0.75–1.10)	3.60	0.006
*T. orientalis* L.,	24	4	Y = 1.45 + 0.80X	1.05 ± 0.19	2.80 (2.45–3.20)	2.40	0.060
*T. orientalis* L.,	48	4	Y = 1.30 + 0.95X	1.30 ± 0.16	2.30 (2.00–2.65)	2.70	0.045
*T. orientalis* L.,	72	4	Y = 1.15 + 1.10X	1.60 ± 0.14	1.80 (1.55–2.10)	3.00	0.030
*T. orientalis* L.,	96	4	Y = 1.00 + 1.25X	1.85 ± 0.13	1.40 (1.20–1.70)	3.20	0.020

**Table 4:** Probit regression analysis of ethanolic plant extracts (24–96 h exposure).

Plant Species	Time (h)	df	Regression Equation (Probit)	Slope ± SE	LC_50_ (%) (95% CI)	χ^2^	*p*-Value
*A. indica* A. Juss.	24	4	Y = 1.35 + 1.15X	1.80 ± 0.13	1.20 (1.05–1.40)	2.95	0.019
*A. indica* A. Juss.	48	4	Y = 1.20 + 1.30X	2.10 ± 0.11	1.00 (0.85–1.20)	3.30	0.010
*A. indica* A. Juss.	72	4	Y = 1.05 + 1.45X	2.35 ± 0.10	0.80 (0.68–0.95)	3.60	0.005
*A. indica* A. Juss.	96	4	Y = 0.90 + 1.65X	2.60 ± 0.08	0.60 (0.50–0.75)	3.85	0.003
*N. rustica* L.,	24	4	Y = 1.40 + 1.05X	1.65 ± 0.14	1.80 (1.60–2.10)	2.85	0.025
*N. rustica* L.,	48	4	Y = 1.25 + 1.20X	1.95 ± 0.12	1.40 (1.20–1.65)	3.10	0.015
*N. rustica* L.,	72	4	Y = 1.10 + 1.35X	2.15 ± 0.10	1.10 (0.95–1.30)	3.45	0.008
*N. rustica* L.,	96	4	Y = 0.95 + 1.50X	2.35 ± 0.09	0.90 (0.75–1.05)	3.70	0.004
*M. azedarach* L.,	24	4	Y = 1.45 + 0.95X	1.40 ± 0.15	2.10 (1.85–2.45)	2.65	0.030
*M. azedarach* L.,	48	4	Y = 1.30 + 1.10X	1.70 ± 0.13	1.70 (1.50–2.00)	2.95	0.020
*M. azedarach* L.,	72	4	Y = 1.15 + 1.25X	2.00 ± 0.11	1.30 (1.10–1.55)	3.25	0.012
*M. azedarach* L.,	96	4	Y = 1.00 + 1.40X	2.25 ± 0.10	1.00 (0.85–1.20)	3.55	0.006
*N. tabacum* L.,	24	4	Y = 1.35 + 1.10X	1.70 ± 0.13	1.60 (1.40–1.90)	2.90	0.015
*N. tabacum* L.,	48	4	Y = 1.20 + 1.25X	2.00 ± 0.11	1.30 (1.10–1.55)	3.20	0.008
*N. tabacum* L.,	72	4	Y = 1.05 + 1.40X	2.25 ± 0.10	1.00 (0.85–1.20)	3.50	0.004
*N. tabacum* L.,	96	4	Y = 0.90 + 1.55X	2.45 ± 0.09	0.80 (0.65–0.95)	3.80	0.002
*T. orientalis* L.,	24	4	Y = 1.50 + 0.90X	1.20 ± 0.16	2.40 (2.10–2.80)	2.55	0.035
*T. orientalis* L.,	48	4	Y = 1.35 + 1.05X	1.50 ± 0.14	1.90 (1.65–2.25)	2.85	0.022
*T. orientalis* L.,	72	4	Y = 1.20 + 1.20X	1.85 ± 0.12	1.50 (1.30–1.75)	3.10	0.014
*T. orientalis* L.,	96	4	Y = 1.05 + 1.35X	2.10 ± 0.11	1.20 (1.00–1.45)	3.35	0.009

## Data Availability

The data presented in this study are available on request from the corresponding author.

## Supplementary Materials

The following supporting information can be downloaded at: https://www.mdpi.com/article/10.3390/insects17070671/s1, Table S1. Oviposition of *C. maculatus* on different hosts in the free-choice test; Table S2. Adult emergence (%) of *C. maculatus* on different hosts in the free-choice test; Table S3. Infestation (%) of *C. maculatus* on different hosts in the free-choice test; Table S4. Weight loss (%) of different hosts in the free-choice test due to *C. maculatus*; Table S5. Mean percent (±SE) of *C. maculatus* females (%) on different hosts in the free-choice test; Table S6. Mean percent (±SE) of *C. maculatus* males (%) on different hosts in the free-choice test; Table S7. Mean percent (±SE) of *C. maculatus* oviposition (eggs/female) after treatment with powders of five plant species; Table S8. Mean (±SE) *C. maculatus* adult emergence (%) after treatment with powders of five plant species; Table S9. Mean (±SE) *C. maculatus* host infestation (%) after treatment with powders of five plant species; Table S10. Mean (±SE) seed weight loss (%) due to *C. maculatus* infestation after treatment with powders of five plant species; Table S11. Mean (±SE) number of *C. maculatus* males (%) after treatment with powders of five plant species; Table S12. Mean (±SE) number of *C. maculatus* females (%) after treatment with powders of five plant species; Table S13. Mean percent (±SE) of *C. maculatus* oviposition (eggs/female) after treatment with aqueous extracts of five plant species; Table S14. Mean (±SE) *C. maculatus* adult emergence (%) after treatment with aqueous extracts of five plant species; Table S15. Mean (±SE) *C. maculatus* host infestation (%) after treatment with aqueous extracts of five plant species; Table S16. Mean (±SE) seed weight loss (%) due to *C. maculatus* infestation after treatment with aqueous extracts of five plant species; Table S17. Mean (±SE) number of *C. maculatus* males (%) after treatment with aqueous extracts of five plant species; Table S18. Mean (±SE) number of *C. maculatus* females (%) after treatment with aqueous extracts of five plant species; Table S19. Mean percent (±SE) of *C. maculatus* oviposition (eggs/female) after treatment with ethanolic extracts of five plant species; Table S20. Mean (±SE) *C. maculatus* adult emergence (%) after treatment with ethanolic extracts of five plant species; Table S21. Mean (±SE) *C. maculatus* host infestation (%) after treatment with ethanolic extracts of five plant species; Table S22. Mean (±SE) seed weight loss (%) due to *C. maculatus* infestation after treatment with ethanolic extracts of five plant species; Table S23. Mean (±SE) number of *C. maculatus* males (%) after treatment with ethanolic extracts of five plant species; Table S24. Mean (±SE) number of *C. maculatus* females (%) after treatment with ethanolic extracts of five plant species; Table S25. Percentage mortality of *C. maculatus*, expressed as mean ± SE, after 24 h of exposure to six graded concentrations of powdered materials derived from five plant species; Table S26. Percentage mortality of *C. maculatus*, expressed as mean ± SE, after 48 h of exposure to six graded concentrations of powdered materials derived from five plant species; Table S27. Percentage mortality of *C. maculatus*, expressed as mean ± SE, after 72 h of exposure to six graded concentrations of powdered materials derived from five plant species; Table S28. Percentage mortality of *C. maculatus*, expressed as mean ± SE, after 96 h of exposure to six graded concentrations of powdered materials derived from five plant species; Table S29. Mean contact-induced mortality (%) (±SE) of *C. maculatus* recorded 24 h after treatment with six concentration levels of aqueous extracts obtained from five plant species; Table S30. Mean contact-induced mortality (%) (±SE) of *C. maculatus* recorded 48 h after treatment with six concentration levels of aqueous extracts obtained from five plant species; Table S31. Mean contact-induced mortality (%) (±SE) of *C. maculatus* recorded 72 h after treatment with six concentration levels of aqueous extracts obtained from five plant species; Table S32. Mean contact-induced mortality (%) (±SE) of *C. maculatus* recorded 96 h after treatment with six concentration levels of aqueous extracts obtained from five plant species; Table S33. Average percentage mortality (±SE) pf *C. maculatus* after 24 h of exposure to six concentration gradients of ethanolic plant extracts prepared from five species; Table S34. Average percentage mortality (±SE) of *C. maculatus* after 48 h of exposure to six concentration gradients of ethanolic plant extracts prepared from five species; Table S35. Average percentage mortality (±SE) of *C. maculatus* after 72 h of exposure to six concentration gradients of ethanolic plant extracts prepared from five species; Table S36. Average percentage mortality (±SE) of *C. maculatus* after 96 h of exposure to six concentration gradients of ethanolic plant extracts prepared from five species.
