# Supplementary material for: Decoupling Host Preference and Performance in Callosobruchus maculatus (Fabricius, 1775): Roles of Seed Biochemistry and Botanical Insecticides in Stored Legumes
Source: Insects. 2026 Jun 26;17(7):671. doi: 10.3390/insects17070671 (PMC13410497; doi:10.3390/insects17070671)
Supplement: Supplementary file 1 [file insects-17-00671-s001.zip › insects-4361008-supplementary.pdf]

Decoupling Host Preference and Performance in *Callosobruchus maculatus* (Fabricius, 1775): Roles of Seed Biochemistry and Botanical Insecticides in Stored Legumes

Rasheed Akbar [1,2], Gul Makai [3], Rehan Kausar [4], Ambreen Ijaz [3], Brekhna Faheem [5], Naseem Rafiq [5], Shehreyar Javed [6], Imtiaz Ali Khan [6], Jibiao Fan [1,*] and Jianfan Sun [7,8,*]

1 College of Animal Science and Technology, Yangzhou University, Yangzhou 225009, China; rasheed.akbar@uoh.edu.pk
2 Department of Entomology, Faculty of Physical and Applied Sciences, The University of Haripur, Haripur 22062, Khyber Pakhtunkhwa, Pakistan
3 Department of Zoology, Sardar Bahadar Khan Women University, Quetta 86400, Balochistan, Pakistan
4 Department of Statistics, Sardar Bahadar Khan Women University, Quetta, 86400, Balochistan, Pakistan;
5 Department of Zoology, Abdul Wali Khan University Mardan, Mardan 23200, Khyber Pakhtunkhwa, Pakistan
6 Department of Entomology, The University of Agriculture Peshawar, Peshawar 22062, Khyber Pakhtunkhwa, Pakistan
7 Institute of Environment and Ecology, School of Environment and Safety Engineering, Jiangsu University, Zhenjiang 212013, China
8 Jiangsu Collaborative Innovation Center of Technology and Material of Water Treatment, Suzhou University of Science and Technology, Suzhou 215009, China
* Correspondence: fanjibiao11@126.com (J.F.); zxsjf@ujs.edu.cn (J.S.)

Table S1. Oviposition of *C. maculatus* on different hosts in free choice test

Source	DF	SS	MS	F	P
Hosts	4	18826.8	4706.70	221	0.0000
Error	15	319.8 21.32			
Total	19	19146.6			

Grand Mean 138.85 CV 3.33

Table S2. Percent emergence of *C. maculatus* on different hosts in free choice test

Source	DF	SS	MS	F	P
Hosts	4	18837.0	4709.25	291	0.0000
Error	15	243.0 16.20			
Total	19	19080.0			

Grand Mean 60.037 CV 6.70

Table S3. Percent infestations of *C. maculatus* on different hosts in free choice test

Source	DF	SS	MS	F	P
Hosts	4	16169.0	4042.26	316	0.0000
Error	15	191.6 12.78			
Total	19	16360.7			

Grand Mean 55.312 CV 6.46

Table S4. Percent seed weight loss of *C. maculatus* on different hosts in free choice test

Source	DF	SS	MS	F	P
Hosts	4	5436.70	1359.18	150	0.0000
Error	15	135.50	9.03		
Total	19	5572.20			

Grand Mean 35.300 CV 8.51

Table S5. Mean percent (± SE) *C. maculatus* male (%) on different hosts in free test

Source	DF	SS	MS	F	P
Hosts	4	2.2193 0.55483	0.09	0.9841	
Error	15	92.3461	6.15641		
Total	19	94.5655			

Grand Mean 50.206 CV 4.94

Table S6. Mean percent (± SE) *C. maculatus* female (%) on different hosts in free choice test

Source	DF	SS	MS	F	P
Hosts	4	3.7698 0.94246	0.16	0.9534	
Error	15	86.2595	5.75063		
Total	19	90.0293			

Grand Mean 49.874 CV 4.81

Table S7. Mean percent (± SE) *C. maculatus* oviposition (eggs/female) after treatment with plant powder extracts of five plant species.

**Source**	**DF**	**SS**	**MS**	**F**	**P**
Replications	3	76	25.2		
Concentrations	5	98671	19734.2	3089.99	0.0000
Plants	4	131655	32913.8	5153.66	0.0000
Plants*concentrations	20	1881	94.0	14.72	0.0000
Error	87	556	6.4		
Total	119	232838			

Grand Mean 192.28 CV 1.31

Table S8. Mean percent (± SE) *C. maculatus* adult emergence (%) after treatment with plant powder extracts of five plant species.

**Source**	**DF**	**SS**	**MS**	**F**	**P**
Replications	3	125.0	41.68		
Plants	4	5683.8	1420.94	445.19	0.0000
Concentrations	5	3005.5	601.11	188.33	0.0000
Plants*concentrations	20	1336.6	66.83	20.94	0.0000
Error	87	277.7	3.19		
Total	119	10428.6			

Grand Mean 71.483 CV 2.50

Table S9. Mean percent (± SE) *C. maculatus* host seed infestation (%) after treatment with plant powder extracts of five plant species.

**Source**	**DF**	**SS**	**MS**	**F**	**P**
Replications	3	10.8	3.59		
Plants	4	7474.5	1868.	4065.06	0.0000
Concentrations	5	5576.3	1115.25	2426.13	0.0000
Plants*concentrations	20	129.2	6.46	14.06	0.0000
Error	87	40.0	0.46		
Total	119	13230.8			

Grand Mean 39.612 CV 1.71

Table S10. Mean percent (± SE) host seed weight loss (%) due to *C. maculatus* infestation after treatment with plant powder extracts of five plant species.

**Source**	**DF**	**SS**	**MS**	**F**	**P**
Replications	3	77.6	25.87		
Plants	4	8120.7	2030.18	190.20	0.0000
Concentration	5	11996.8	2399.35	224.78	0.0000
Plants*concentration	20	952.9	47.65	4.46	0.0000
Error	87	928.7	10.67		
Total	119	22076.6			

Grand Mean 24.281 CV 7.46

Table S11. Mean percent (± SE) *C. maculatus* male (%) after treatment with plant powder extracts of five plant species.

**Source**	**DF**	**SS**	**MS**	**F**	**P**
Replications	3	2.1045	0.70151		
Plants	4	1.3256	0.33140	0.99	0.4189
Concentration	5	0.5842	0.11684	0.35	0.8822
Plants * Concentration	20	6.8076	0.34038	1.01	0.4551
Error	87	29.2059	0.33570		
Total	119	40.0278			

Grand Mean 50.264 CV 1.15

Table S12. Mean percent (± SE) *C. maculatus* female (%) after treatment with plant powders extracts of five plant species.

**Source**	**DF**	**SS**	**MS**	**F**	**P**
Replications	3	2.1064	0.70212		
Plants	4	1.3264	0.33159	0.99	0.4184
Concentrations	5	0.5864	0.11727	0.35	0.8813
Plants * concentrations	20	6.8068	0.34034	1.01	0.4548
Error	87	29.1947	0.33557		
Total	119	40.0206			

Grand Mean 49.736 CV 1.16

Table S13. Mean percent (± SE) *C. maculatus* oviposition (eggs/female) after treatment with plant aqueous extracts of five plant species.

**Source**	**DF**	**SS**	**MS**	**F**	**P**
Replications	3	153	51.0		
Concentrations	5	83979	16795.8	1011.84	0.0000
Plants	4	88350	22087.6	1330.64	0.0000
Plants*concentrations	20	315	15.7	0.95	0.5313
Error	87	1444	16.6		
Total	119	174241			

Grand Mean 168.30 CV 2.42

Table S14. Mean percent (± SE) *C. maculatus* adult emergence (%) after treatment with aqueous extracts of five plant species.

**Source**	**DF**	**SS**	**MS**	**F**	**P**
Replications	3	41.39	13.796		
Plants	4	934.19	233.547	38.52	0.0000
Concentration	5	1333.34	266.667	43.99	0.0000
Plants* concentration	20	638.00	31.900	5.26	0.0000
Error	87	527.43	6.062		
Total	119	3474.35			

Grand Mean 77.661 CV 3.17

Table S15. Mean percent (± SE) *C. maculatus* host seed infestation (%) after treatment with aqueous extracts of five plant species.

**Source**	**DF**	**SS**	**MS**	**F**	**P**
Replications	3	3.5	1.17		
Plants	4	4913.8	1228.46	1276.93	0.0000
Concentration	5	5659.5	1131.90	1176.57	0.0000
Plants * concentration	20	47.0	2.35	2.44	0.0023
Error	87	83.7	0.96		
Total	119	10707.6			

Grand Mean 36.004 CV 2.72

Table S16. Mean percent (± SE) host seed weight loss (%) due to *C. maculatus* infestation after treatment with aqueous extracts of five plant species.

**Source**	**DF**	**SS**	**MS**	**F**	**P**
Replications	3	37.4	12.46		
Plants	4	10258.2	2564.56	630.89	0.0000
Concentration	5	5091.2	1018.25	250.49	0.0000
Plants *concentration	20	187.1	9.36	2.30	0.0042
Error	87	353.7	4.06		
Total	119	15927.6			

Grand Mean 25.517 CV 3.90

Table S17. Mean percent (± SE) *C. maculatus* male (%) after treatment with aqueous extracts of five plant species.

**Source**	**DF**	**SS**	**MS**	**F**	**P**
Replications	3	1.2834	0.42779		
Plants	4	0.6639	0.16598	0.35	0.8420
Concentration	5	1.0099	0.20198	0.43	0.8277
Plants * concentration	20	6.0040	0.30020	0.64	0.8741
Error	87	41.0201	0.47150		
Total	119	49.9813			

Grand Mean 50.159 CV 1.37

Table S18. Mean percent (± SE) *C. maculatus* females (%) after treatment with aqueous extracts of five plant species.

**Source**	**DF**	**SS**	**MS**	**F**	**P**
Replications	3	2.5443	0.84809		
Plants	4	4.4015	1.10037	2.27	0.0683
Concentration	5	2.3797	0.47594	0.98	0.4341
Plants * concentration	20	9.6728	0.48364	1.00	0.4743
Error	87	42.2099	0.48517		
Total	119	61.2082			

Grand Mean 49.725 CV 1.40

Table S19. Mean percent (± SE) *C. maculatus* oviposition (eggs/female) after treatment with plant ethanolic extracts of five plant species.

**Source**	**DF**	**SS**	**MS**	**F**	**P**
Replications	3	37.7	12.57		
Concentrations	5	11287.0	2257.40	1032.02	0.0000
Plants	4	16580.5	4145.13	1895.04	0.0000
Plants*concentrations	20	246.2	12.31	5.63	0.0000
Error	87	190.3	2.19		
Total	119	28341.7			

Grand Mean 104.95 CV 1.41

Table S20. Mean (± SE) *C. maculatus* adult emergence (%) after treatment with plant ethanolic extracts of five plant species,

**Source**	**DF**	**SS**	**MS**	**F**	**P**
Replications	3	9.3	3.12		
Plants	4	8675.0	2168.75	778.42	0.0000
Concentrations	5	6543.7	1308.73	469.74	0.0000
Plants* concentrations	20	331.5	16.57	5.95	0.0000
Error	87	242.4	2.79		
Total	119	15801.9			

Grand Mean 69.086 CV 2.42

Table S21. Mean (± SE) *C. maculatus* host infestation (%) after treatment with plant ethanolic extracts of five plant species.

**Source**	**DF**	**SS**	**MS**	**F**	**P**
Replications	3	2.62	0.872		
Plants	4	1184.43	296.108	1344.56	0.0000
Concentrations	5	1699.28	339.856	1543.21	0.0000
Plants*concentrations	20	11.74	0.587	2.67	0.0009
Error	87	19.16	0.220		
Total	119	2917.23			

Grand Mean 21.300 CV 2.20

Table S22. Mean (± SE) seed weight loss (%) due to *C. maculatus* infestation after treatment with plant ethanolic extracts of five plant species.

**Source**	**DF**	**SS**	**MS**	**F**	**P**
Replications	3	2.17	0.72		
Plants	4	4836.38	1209.09	984.83	0.0000
Concentrations	5	1955.19	391.04	318.51	0.0000
Plants*concentrations	20	38.84	1.94	1.58	0.0758
Error	87	106.81	1.23		
Total	119	6939.38			

Grand Mean 19.156 CV 5.78

Table S23. Mean (± SE) *C. maculatus* male (%) after treatment with plant ethanolic extracts of five plant species.

**Source**	**DF**	**SS**	**MS**	**F**	**P**
Replications	3	2.0799	0.69329		
Plants	4	0.5826	0.14564	0.49	0.7428
Concentrations	5	0.9600	0.19201	0.65	0.6649
Plants*concentrations	20	1.7927	0.08963	0.30	0.9982
Error	87	25.8421	0.29704		
Total	119	31.2572			

Grand Mean 50.094 CV 1.09

Table S24. Mean (± SE) *C. maculatus* female (%) after treatment with plant ethanolic extracts of five plant species.

**Source**	**DF**	**SS**	**MS**	**F**	**P**
Replications	3	3.4930	1.16434		
Plants	4	1.3733	0.34334	0.83	0.5078
Concentrations	5	0.9949	0.19898	0.48	0.7882
Plants*concentrations	20	1.2236	0.06118	0.15	1.0000
Error	87	35.8564	0.41214		
Total	119	42.9412			

Grand Mean 49.915 CV 1.29

Table S25: Percentage mortality of *C. maculatus*, expressed as mean ± SE, after 24 h exposure to six graded concentrations of powdered materials derived from five plant species.

**Source**	**DF**	**SS**	**MS**	**F**	**P**
Replications	3	180.00	60.000		
Plants	4	970.00	242.500	9.29	0.0000
Concentrations	5	2996.67	599.333	22.97	0.0000
Plants*concentrations	20	170.00	8.500	0.33	0.9969
Error	87	2270.00	26.092		
Total	119	6586.67			

Grand Mean 10.333 CV 4.43

Table S26. Percentage mortality of *C. maculatus*, expressed as mean ± SE, after 48 h exposure to six graded concentrations of powdered materials derived from five plant species.

**Source**	**DF**	**SS**	**MS**	**F**	**P**
Replications	3	170.6	56.86		
Plants	4	1784.1	446.01	12.24	0.0000
Concentrations	5	6996.7	1399.33	38.41	0.0000
Plants*concentrations	20	269.2	13.46	0.37	0.9931
Error	87	3169.4	36.43		
Total	119	12390.0			

Grand Mean 23.983 CV 5.17

Table S27. Percentage mortality of *C. maculatus*, expressed as mean ± SE, after 72 h exposure to six graded concentrations of powdered materials derived from five plant species.

**Source**	**DF**	**SS**	**MS**	**F**	**P**
Replications	3	182.0	60.68		
Plants	4	7411.9	1852.99	76.96	0.0000
Concentrations	5	29556.8	5911.36	245.52	0.0000
Plants*concentrations	20	438.8	21.94	0.91	0.5742
Error	87	2094.7	24.08		
Total	119	39684.3			

Grand Mean 47.675 CV 10.29

Table S28. Percentage mortality of *C. maculatus*, expressed as mean ± SE, after 96 h exposure to six graded concentrations of powdered materials derived from five plant species.

**Source**	**DF**	**SS**	**MS**	**F**	**P**
Replications	3	60.0	20.01		
Plants	4	8892.2	2223.04	117.36	0.0000
Concentrations	5	25248.5	5049.69	266.59	0.0000
Plants*concentrations	20	2808.5	140.43	7.41	0.0000
Error	87	1648.0	18.94		
Total	119	38657.2			

Grand Mean 80.917 CV 5.38

Table S29. Mean contact-induced mortality (%) (± SE) of *C. maculatus* recorded 24 h after treatment with six concentration levels of aqueous extracts obtained from five plant species.

**Source**	**DF**	**SS**	**MS**	**F**	**P**
Replications	3	153.3	51.11		
Plants	4	2220.0	555.00	10.62	0.0000
Concentrations	5	6146.7	1229.33	23.52	0.0000
Plants*concentrations	20	120.0	6.00	0.11	1.0000
Error	87	4546.7	52.26		
Total	119	13186.7			

Grand Mean 26.333 CV 2.45

Table S30. Mean contact-induced mortality (%) (± SE) of *C. maculatus* recorded 48 h after treatment with six concentration levels of aqueous extracts obtained from five plant species.

**Source**	**DF**	**SS**	**MS**	**F**	**P**
Replications	3	118.5	39.49		
Plants	4	1408.9	352.22	4.92	0.0013
Concentrations	5	10673.6	2134.71	29.84	0.0000
Plants*concentrations	20	1265.9	63.30	0.88	0.6059
Error	87	6223.0	71.53		
Total	119	19689.9			

Grand Mean 43.633 CV 3.38

Table S31. Mean contact-induced mortality (%) (± SE) of *C. maculatus* recorded 72 h after treatment with six concentration levels of aqueous extracts obtained from five plant species.

**Source**	**DF**	**SS**	**MS**	**F**	**P**
Replications	3	670.7	223.57		
Plants	4	4663.2	1165.80	16.15	0.0000
Concentrations	5	28591.0	5718.19	79.23	0.0000
Plants*concentrations	20	926.3	46.31	0.64	0.8699
Error	87	6278.8	72.17		
Total	119	41130.0			

Grand Mean 62.683 CV 8.55

Table S32. Mean contact-induced mortality (%) (± SE) of *C. maculatus* recorded 96 h after treatment with six concentration levels of aqueous extracts obtained from five plant species.

**Source**	**DF**	**SS**	**MS**	**F**	**P**
Replications	3	280.8	93.59		
Plants	4	21749.9	5437.47	89.27	0.0000
Concentrations	5	9112.7	1822.55	29.92	0.0000
Plants*concentrations	20	1909.7	95.49	1.57	0.0798
Error	87	5299.5	60.91		
Total	119	38352.6			

Grand Mean 83.608 CV 9.33

Table S33. Average percentage mortality remaining (± SE) in *C. maculatus* after 24 h of exposure to six concentration gradients of ethanolic plant extracts prepared from five species

**Source**	**DF**	**SS**	**MS**	**F**	**P**
Replications	3	166.67	55.556		
Plants	4	1255.00	313.750	13.10	0.0000
Concentrations	5	2160.00	432.000	18.04	0.0000
Plants*concentrations	20	215.00	10.750	0.45	0.9777
Error	87	2083.33	23.946		
Total	119	5880.00			

Grand Mean 9.0000 CV 6.37

Table S34. Average percentage mortality remaining (± SE) in *C. maculatus* after 48 h of exposure to six concentration gradients of ethanolic plant extracts prepared from five species

**Source**	**DF**	**SS**	**MS**	**F**	**P**
Replications	3	89.17	29.72		
Plants	4	986.67	246.67	10.29	0.0000
Concentration	5	5204.17	1040.83	43.41	0.0000
Plants *concentration	20	133.33	6.67	0.28	0.9990
Error	87	2085.83	23.98		
Total	119	8499.17			

Grand Mean 15.917 CV 3.76

Table S35. Average percentage mortality remaining (± SE) in *C. maculatus* after 72 h of exposure to six concentration gradients of ethanolic plant extracts prepared from five species.

**Source**	**DF**	**SS**	**MS**	**F**	**P**
Replications	3	110.3 36.76			
Plants	4	3414.1	853.53 18.18	0.0000	
Concentrations	5	15928.5	3185.71	67.86	0.0000
Plants*concentrations	20	196.6	9.83	0.21	0.9999
Error	87	4084.5	46.95		
Total	119	23734.0			

Grand Mean 31.842 CV 2.52

Table S36. Average percentage mortality remaining (± SE) in *C. maculatus* after 96 h of exposure to six concentration gradients of ethanolic plant extracts prepared from five species

**Source**	**DF**	**SS**	**MS**	**F**	**P**
Replications	3	345.7	115.23		
Plants	4	11338.6	2834.65	62.97	0.0000
Concentrations	5	28952.9	5790.57	128.63 0.0000	
Plants*concentrations	20	218.4 10.92	0.24	0.9996	
Error	87	3916.6	45.02		
Total	119	44772.1			

Grand Mean 60.625 CV 5.07
